# Discrimination: a health hazard for people from refugee and asylum-seeking backgrounds resettled in Australia

**DOI:** 10.1186/s12889-019-8068-3

**Published:** 2020-01-28

**Authors:** Anna Ziersch, Clemence Due, Moira Walsh

**Affiliations:** 1 Southgate Institute for Health, Society and Equity, Flinders University, GPO Box 2100, Adelaide, SA 5001 Australia; 2 School of Psychology Faculty of Health and Medical Sciences, University of Adelaide, Adelaide, SA 5005 Australia

**Keywords:** Australia, Discrimination, Refugee, Asylum seeker, Mental health, Responses

## Abstract

**Background:**

Research has shown that discrimination is harmful to health, but there is relatively little known about discrimination experienced by people from refugee and asylum-seeking backgrounds in resettlement countries and associated health effects. This qualitative-focused mixed methods paper reports on discrimination experienced by refugees and asylum seekers, responses to discrimination, and impacts on health.

**Methods:**

As part of a broader study of housing, social inclusion and health, surveys were completed by 423 adult refugees and asylum seekers living in South Australia who had been in Australia for up to 7 years. The survey included questions on discrimination based on skin colour, ethnicity and religion, as well as questions on hope, trust, belonging, sense of control and health (including the SF-8). Semi-structured interviews were conducted with 65 survey participants, purposively sampled by visa status, continent and gender, further exploring experiences of discrimination. These and survey open-ended responses were analysed thematically.

**Results:**

Twenty-two percent of survey participants reported experiences of discrimination since arriving in Australia (14% in the last year), and 90% of these felt that discrimination had harmed their health. Key settings of discrimination were public transport, within the neighbourhood, and in relation to employment. Those who reported discrimination had significantly worse mental health (*p* < .000) but not physical health. Discrimination was also associated with less sense of belonging (*p* = .001), lower levels of trust (*p* = .038), reduced sense of control (*p* = .012) and less hope (*p* = .006). Incidents described in interviews and the open-ended survey responses included incivility, physical assault, and denial of services, experienced across intersecting characteristics of race/ethnicity, religion, gender and visa status. Responses to discrimination spanned affective, cognitive and behavioural dimensions, ranging across types of experience, participant characteristics and context, with most individuals reporting multiple response types. While some of the responses were reported by participants as protective of health, participants’ reflections indicated significant negative impacts on mental health in particular.

**Conclusion:**

Discrimination featured in the resettlement experiences of a significant number of refugees and asylum seekers, with participants reporting clear negative impacts on mental health. Addressing discrimination is a key resettlement and health issue requiring urgent action.

## Background

People from refugee and asylum-seeking backgrounds have compounding risks for poor mental and physical health [1–3]. Discrimination is a well-known social determinant of health, and so experiences of discrimination in countries of resettlement have the potential to exacerbate negative health and wellbeing (hereafter ‘health’) outcomes. However, little research has examined this specifically for refugees and asylum seekers. The small body of existing research suggests that refugees and asylum seekers face discrimination in a range of resettlement areas including housing, education, neighbourhoods and health care access, with likely health consequences. However, responses to discrimination and the precise pathways between discrimination and health remain under-explored for this population.

This paper reports on a mixed-methods study of experiences of, and responses to, discrimination, and the associated health impacts. The paper draws on qualitative data from interviews, supplemented by quantitative survey data, from a study with refugees and asylum seekers living in Adelaide, Australia. The extent and nature of discrimination experienced, how participants made sense of and responded to these experiences, and the impacts on health are examined.

### A note on terminology

Refugees are defined as people who meet the criteria for refugee status according to the United Nations High Commissioner for Refugees (UNHCR) and asylum seekers are defined as those awaiting their claims to refugee status to be determined [4], but at times by criteria outlined by specific countries [5]. In this paper for brevity we use ‘refugee’ and ‘asylum seeker’ but acknowledge the complex identities subsumed under these terms. We consider race, ethnicity and culture to be separate but related constructs, which are often conflated [6]. Importantly, we do not use ‘race’ to refer to biological aspects, but rather race/ethnicity is used to indicate a person’s affiliation with a particular ethnic group, often but not always associated with country of origin, while culture refers to shared systems of meaning making, which are learnt and shared across generations and sometimes includes religious affiliation [7]. In relation to health we draw on the World Health Organization definition of health as “a state of complete physical, mental and social wellbeing and not merely the absence of disease or infirmity” [8](p.g.100).

### Discrimination and health

Discrimination is “a socially structured and sanctioned phenomenon, justified by ideology and expressed in interactions among and between individuals and institutions, that maintains privileges for members of dominant groups at the cost of deprivation for others” [9](pg. 650). Discrimination can be both overt and covert, and can occur at an institutional level (practices, policies or processes that reproduce and/or maintain avoidable inequalities across groups), an interpersonal level (interactions between individuals) and an internalised level (internalised ideologies, beliefs or attitudes about the inferiority of own group) [9–12]. Discrimination can range from physical violence and direct threats and insults, to systemic limitations around access to resources such as housing, employment and education [6, 11].

Key reviews and meta-analyses underscore the negative impact of discrimination on both mental and physical health, across a range of characteristics such as race/ethnicity, gender and age (e.g. [6, 9, 12–21]). In addition to directly experiencing discrimination, witnessing or experiencing vicarious discrimination can also affect health [22–24]. These reviews highlight the complex and multiple pathways through which discrimination can affect health, including physiological responses, internalisation of negative stereotypes, damaging coping behaviours (such as drug and alcohol abuse), physical violence, and unequal access to resources.

An intersectional approach to discrimination views privilege, oppression and disadvantage as the result of the combined effects of social identities/ categories (e.g., race/ethnicity, migration status, religion, gender), which intersect within interrelated structures of power [25–28]. Experiences of discrimination are thus shaped by multiple, intersecting categories, and the impacts on health are interactional and multiplicative [28–30]. We focus broadly on discrimination in this paper (rather than racial discrimination or racism only), in order to consider these overlapping categories, and draw on intersectionality, using a process-centered approach, taking account of the impacts of discrimination at the location of various combinations of social categories on health [31].

### Refugees, asylum seekers and discrimination

Humanitarian migration is contested in public discourse in Australia (and internationally), with debates concerning multiculturalism, assimilation and integration, as well as increased levels of Islamophobia [32–35], and progressively punitive policies aimed at asylum seekers, particularly those who arrive by boat [36]. In this context, experiences of discrimination feature prominently in the resettlement accounts of refugees and asylum seekers in Australia and abroad, including in employment, access to social services, and within neighbourhoods (e.g. [37–44]). Moreover, institutional discrimination is evident in government policies, particularly through restrictions on services for those on temporary visas [36].

The limited research directly examining the link between discrimination and health for this population suggests that discrimination may be particularly damaging for refugees and asylum seekers, compounding pre-migration trauma and persecution [40, 43–56]. Discrimination can also affect resettlement and integration, indirectly impacting on health [57].

### Responses to discrimination and relevance for health outcomes

Examining how people respond to discrimination helps to further understand - and potentially interrupt - the pathways through which discrimination harms health [58, 59]. However, it is important to note that this does not place a burden of responsibility on those who experience discrimination, which remains a systemic issue requiring urgent action.

Previous research has framed responses to discrimination as affective, cognitive and behavioural [11, 58], showing how individual responses may buffer negative impacts on health. Responses have also been categorised as active (e.g. lodging a complaint) or passive (e.g. ignoring) with evidence that active responses that increase agency may be more protective [9, 11, 13, 58]. However, there are some inconsistencies in this research, and evidence that there can be a ‘cost of coping’ in terms of cognitive load, reduced opportunities, and health impacts [11].

There is very limited research examining responses to discrimination by refugees and asylum seekers, and how these responses influence the impact of discrimination on health. There is some evidence of avoidance as a key strategy – for example, in choosing where to live or avoiding social encounters [60], though this has not been examined specifically in relation to health impacts. Verkuyten and Nekuee found that strong ethnic identification influenced coping strategies amongst Iranian refugees in the Netherlands [61]. Noh et al., examining experiences of discrimination for a sample of South East Asian refugees in Canada, also highlight the importance of cultural norms and social contexts in examining the health protective effects of ways of responding to discrimination [51]. For example, they argue that emotional-focused coping (for example acting with ‘forbearance’) can be more protective of health than problem-focused coping such as an act of confrontation, when this approach aligns with cultural norms [51]. Fozdar and Torezani suggest that some refugees may respond by considering discrimination to be an individual phenomenon, rather than systematic, and therefore less damaging. However, this research is in its infancy and there is a need to further understand responses to discrimination for refugees and asylum seekers in countries of resettlement.

### Aims and research questions

Our research aimed to explore experiences of and responses to discrimination for refugees and asylum seekers. Specifically, we examined: 1) To what extent was discrimination reported, what was the nature of reported experiences, and how were they framed by participants? 2) What were participants’ responses to experiences of discrimination?; and 3) How were experiences of, and responses to, discrimination linked to health?

## Methods

This paper draws on findings from a larger study on the impacts of housing, neighbourhood and social inclusion on health for refugees and asylum seekers [62, 63]. The main focus of this paper is on the qualitative data from the study. However, we provide a snapshot of the quantitative data to supplement and contexualise findings.

### Procedure

Ethics approval was obtained from the Flinders University Social and Behavioural Ethics Committee (Project 6723) and the researchers paid particular attention to potential issues of coercion and informed consent, power imbalances between researchers and participants, as well as concerns about confidentiality and anonymity [64, 65]. Project documentation was translated into key languages, and interpreters were available. The project was conducted in partnership with a project reference group and a refugee and asylum seeker advisory group. Informed consent was gained from all participants prior to participation. Data was collected from June 2015–January 2017.

Participants were refugees and asylum seekers aged 18 and above, living in Australia for 7 years or less, currently resident in South Australia. Data collection involved both a survey with closed and open-ended questions (Additional file 1) and semi-structured in-depth interviews (Additional file 2). Survey participants (*N* = 423) were recruited through organisations, community groups and passive snowball sampling. Semi-structured interviews were conducted with a subset of 65 survey participants who indicated their interest in participating in an interview, purposively sampled for cultural background, visa status and gender. The interviews took place at a venue of the participants’ choosing and lasted up to 70 min (average of 32 min), with an interpreter if the participant elected. Interviews were conducted by 4 female researchers, none of whom were migrants – the potential relevance of this for disclosure of discrimination is discussed below.

In this paper, we use pseudonyms and include visa status (permanent visa (PV) and temporary visa (TV)), continent (Middle East, Africa, South East (SE) Asia) and gender (where this is not clear from the context), for direct quotes.

### Measures and data analysis

A single item discrimination measure from the Scanlon Foundation was used [66], in order to facilitate a comparison of prevalence rates with the annual Scanlon Foundation survey of the Australian general population: ‘Have you experienced discrimination or been treated unfairly in Australia because of your skin colour, ethnic origin or religion?’, with response categories: ‘yes, more than 12 months ago’, ‘yes, within the last 12 months’, ‘no’, and list of possible settings provided for those who ticked “yes”. Participants were also asked to share their experiences of discrimination in an open-ended response, and to rate the extent to which they felt this discrimination had “…affected your health and wellbeing” (not at all, slightly, moderately, quite a bit, a great deal).

The survey also included items for trust (“to what extent do you agree that most people can be trusted”) and belonging (“to what extent do you have a sense of belonging in Australia”) (not at all, only slightly, to a moderate extent, and to a great extent – dichotomised for analysis into not at all/only slightly versus moderate/great extent). Participants were also asked their level of agreement in relation to control (“I feel in control of my life”) and hope (“I feel hopeful about the future”) (disagree a lot, disagree a bit, don’t agree or disagree, agree a bit, and agree a lot – categorised for analysis to agree a bit/a lot v disagree a lot/a bit/don’t agree or disagree).

Health was measured using the Short Form-8 (SF-8) health measure, which returns a mental health composite score (MCS) and a physical health composite score (PCS).

The survey data was analysed with IBM SPSS Version 23. Univariate analysis was undertaken using chi-square tests and independent samples t-tests.

Interview questions covered a range of topics including questions about housing, neighbourhood and health, social and civic participation, and supports in Australia. There were a number of questions that explicitly asked about experiences of discrimination, responses and whether participants thought that these experiences had an impact on their health. Participants also discussed discrimination experiences unprompted in other sections of the interview.

The open-ended survey and interview data were thematically analysed using the 5 stage framework approach [67]: familiarisation with the data (reading and re-reading transcripts); development of a thematic framework (done inductively and iteratively from the data); indexing (coding with NVivo Version 10 (QSR International; 2012), with a subset double coded by the research team and any inconsistencies resolved with discussion); charting (thematic matrices charting each participant against the emergent themes); and mapping and interpretation (where experiences of discrimination, responses and health impacts are outlined). The findings were discussed with the project reference and advisory groups (member-checking).

### Participants

423 people completed the survey (Table 1). 53% of participants were female, 89% under 50 years old, and almost three quarters were on permanent refugee visas. Over half came from the Middle East, around a third from Africa and the remainder from SE Asia. For reported religious identification the largest group practiced Islam, followed by Christianity.

**Table 1:** Survey participant characteristics

	Total (*N* = 423), (%)	Male (*N* = 188), (%)	Female (*N* = 215)*, (%)
*Age*			
18–29	173 (40.9)	78 (41.5)	87 (40.5)
30–49	202 (47.8)	86 (45.7)	109 (50.7)
50+	44 (10.4)	23 (12.2)	16 (7.4)
	4 missing		
*Visa*			
Refugee	296 (70)	121 (64.4)	161 (74.9)
Asylum Seeker	113 (26.7)	60 (31.9)	49 (22.8)
	14 missing		
*Time in Australia*			
≤ 6 months	62 (14.7)	28 (14.9)	27 (12.6)
7 months- < 2 years	103 (24.3)	44 (23.4)	56 (26.0)
2- < 5 years	190 (44.9)	85 (45.2)	98 (45.6)
5+ years	66 (15.6)	30 (16.0)	34 (15.8)
	2 missing		
*Continent*			
Middle East	221 (52.2)	95 (50.5)	112 (52.1)
Africa	137 (32.4)	64 (34.0)	68 (31.6)
Southeast Asia	57 (13.5)	24 (12.8)	32 (14.9)
	8 missing		
*Religion*			
Christian	141 (33.3)	61 (32.4)	75 (34.9)
Islam	195 (46.1)	82 (43.6)	103 (47.9)
Other	49 (11.6)	24 (12.8)	23 (10.7)
None	33 (7.8)	20 (10.6)	12 (5.6)
	5 missing		
*Discrimination*			
Yes	91 (21.5)	38 (20.2)	52 (24.2)
No	316 (74.7)	144 (76.6)	157 (73.0)
	16 missing		

*20 missing gender

Interview participants comprised 34 refugees with permanent protection visas (PV) (15 women and 19 men; 12 from Africa, 12 from the Middle East and 10 from SE Asia) and 31 asylum seekers with temporary visas (TV) (13 women and 18 men, 30 from the Middle East and one from SE Asia, reflecting the profile of asylum seekers in Australia).

## Results

### Results from the quantitative survey

91 participants (22%) said that they had experienced discrimination since being in Australia. Of these, 55 (60%) reported that this had occurred within the last year, and 38 (42%) more than a year ago, with 2 people reporting discrimination in both timeframes. The main places where discrimination had occurred were on public transport (*N* = 30, 33%), within the neighbourhood (*N* = 27, 30%) and in employment (*N* = 21, 23%). Other settings were in services (e.g. shops and taxis) and housing (both *N* = 17, 19%), education (*N* = 13, 14%), health (*N* = 10, 11%), policing (*N* = 6, 7%), financial (*N* = 3, 3%) and 12 reported ‘other’ settings.

We examined experiences of discrimination by the participant variables (Table 2). We found significant differences by time in Australia (with higher rates of discrimination with longer resettlement period), continent (participants from Africa and the Middle East reported higher rates of discrimination than those from SE Asia, (marginal)) visa status (asylum seekers reported higher rates than refugees) and religion (participants with no-religion reported the highest rates, followed by Christian, Muslim and then other - notably, 29 of the 33 participants who reported no religion were from the Middle East, and 21 were asylum seekers). Further analysis considered the sample as a whole due to sample size constraints.

**Table 2:** Reported discrimination by demographic variables

		Discrimination N(%)	No discrimination N(%)
Gender	Male	38 (20.9)	144 (79.1)
	Female	52 (24.9)	157 (75.1)
			*p* = 0.348
Age (years)	18–29	32 (19.5)	132 (80.5)
	30–49	48 (24.5)	148 (75.5)
	50+	8 (18.6)	35 (81.4)
			*p* = 0.451
Visa status	Refugee	50 (17.4)	238 (82.6)
	Asylum Seeker	36 (33.6)	71 (66.4)
			*p* = <0.001
Time in Australia	≥6 months	5 (8.6)	53 (91.4)
	7 months- < 2 yrs	16 (15.8)	85 (84.2)
	2- < 5 yrs	51 (28.0)	131 (72.0)
	5 + years	19 (29.2)	46 (70.8)
			*p* = 0.003
Continent	Middle East	46 (22.1)	162 (77.9)
	Africa	37 (27.2)	99 (72.8)
	South East Asia	6 (10.9)	49 (89.1)
			*p* = 0.050
Religion	Christian	41 (29.7)	97 (70.3)
	Muslim	33 (17.6)	155 (82.4)
	Other	4 (8.5)	43 (91.5)
	No religion	13 (40.6)	19 (59.4)
			*p* = <0.001

Of those who had experienced discrimination, 90% (*N* = 77, 5 missing) thought that it damaged their health. We found a significant difference in mean MCS scores (t = −.404, df 383, *p* < .0001) for those reporting discrimination (*M* = 38.84, *SD* = 10.83) compared to those who had not (*M* = 43.70, *SD* = 9.64), but no significant difference for PCS scores (t = 0.16, df 383, *p* = .988) between those who reported discrimination (*M* = 46.17, *SD* = 9.66) and those who had not (*M* = 46.16, *SD* = 8.90).

Those who had experienced discrimination also reported less sense of belonging (82% vs 65% felt they belonged, χ^2^ = 11.90, df = 1, *p* = .001), less hope for the future (79% vs 65% felt hopeful about the future, χ^2^ = 7.56, df = 1, *p* = .006), less sense of control (65% vs 50% felt in control of their life, χ^2^ = 6.26, df = 1, *p* = .012) and lower levels of trust (64% vs 52% trusted people in general, χ^2^ = 4.32, df = 1, *p* = .038).

### Results from the interviews and open-ended survey responses

#### Experiences of discrimination

31 of the 65 interview participants described personal experiences of discrimination in Australia, and seven more gave accounts of others (friends, relatives, community members) who had (hereafter ‘vicarious discrimination’). Importantly, eight participants who provided accounts of discrimination in interviews, had indicated “no” to discrimination in the survey.

Discrimination reported in both the survey and interviews spanned interpersonal experiences of incivility through to threats and physical violence and institutional discrimination. Discrimination seemed to particularly occur at the intersections of immigration status, race/ethnicity, religion and gender.

##### Incivility

Participants – most notably from the Middle East and Africa - described a range of experiences of incivility, which they perceived as discriminatory. For instance, being spoken to ‘differently’, ignored or overlooked, and treated in an ‘unfriendly’ manner. Others described more explicit experiences of discriminatory incivility on the basis of their ethnicity and migration status, with various manifestations of the phrase “go back where you came from”, in particular on public transport and in shopping centres. For example, Kasra, a Middle Eastern asylum seeker, described being asked by a stranger on the bus “*why did you come by boat?”* while having a diaper thrown at her.

Other women from the Middle East described experiences of discrimination based on the intersection of religion (or culture, given the complex interplay between religion and culture; e.g. [32]) and gender such as wearing a hijab. These women described unwelcome staring and verbal attacks in the street and at the local park, for example Geeti recounted “*sometimes when cars pass by me and they see that I wear headscarf, they insult me…that hurts a lot”* (Middle East, PV). Moreover, for Naeva, a Muslim woman from Africa, the intersecting categories of skin colour, religion and gender were salient in a verbal attack from a stranger in the street. Her daughter, who was also present during the attack, translated:

*There was a lady that was just walking past. Like I don’t know if she was having a bad day or anything [but] she saw black people plus Muslim people and she just started going off and off at us for no reason […] I was so shocked.*

Discrimination based on intersecting social categories also featured in the accounts of several young Middle Eastern men with religion, ethnicity and country of origin being associated with links to terrorism. For example, Janan a male asylum seeker from the Middle East described:

*When I talk about myself, that I’m from Afghanistan, [people] get different with me. […] most of the people don’t like Afghani [...] Maybe they think [we are] terrorist or something*.

Some experiences of incivility had elements of explicit threat including within neighbourhoods. For example, Yatindra (SE Asia, PV), described finding a note in her letterbox saying “*‘you go back’”,* Eskandar (Middle East, TV) detailed the constant abuse he suffered from a neighbour, who repeatedly said: “‘*go back to your fucking country where you fucking came from’”*, and Rachel (SE Asia, PV) recounted a neighbour who would regularly thump on her front door while shouting insults. Rachel didn’t understand exactly what she was saying but said: *“maybe she thought we were Muslims*”. Other participants likewise said that though they didn’t necessarily understand what was being said – they registered the threatening tone.

Some participants found it more difficult to explicitly label their experiences of incivility as discrimination. For example, in describing incidents where fellow passengers on the bus refused to share a seat with him, Solomon asked “*Is it because I’m black?”* (Africa, PV). Other respondents from Africa similarly emphasised the potentially “hidden” nature of discrimination in Australia: “*so I didn’t say there is not any discrimination, but the type of discrimination is masked discrimination”* (Samson, Africa, Male, PV).

Participants also recounted experiences of vicarious discrimination. For example, Banou (Africa, PV) indicated that her children had experienced discrimination at school: “*some people there, telling them that their skin colour is not shining enough. So the children told me this information”*, and Naeva’s (Africa, PV) daughters were present when their mother experienced racial abuse as described previously. Similarly, Nikta (Middle East, female, TV) reported:

*The lady started [C word] and she punched on the table and told him* [my friend] *‘I think Nauru* [which houses an offshore detention centre used by Australia] *is not enough for you because you are robbing our money there. We have many empty prisons in South Australia, that you should be there forever*

##### Physical assault

Several participants described experiences of discrimination that were violent, typically at the intersection of ethnicity/race, religion and gender. For example, Naweed a Middle Eastern asylum seeker recounted being physically assaulted after he confronted a man who had verbally abused his wife, who was wearing a headscarf. Davoud (Middle East, male, TV) also described being slapped while waiting for a bus, and Sarina (Middle East, female, TV) said: *“some people in the bus, single boys, they start fighting to my husband and they hit him*.” Moreover, Adeeb (Middle East, TV) described being physically and verbally assaulted in his neighbourhood on the basis of his ethnicity/culture, with one perpetrator saying: “*We hate the Afghani guys. When you Afghani guys came to Australia, they make a lot of problems.”*

##### Systemic discrimination

Participants also discussed systemic discrimination, such as in accessing housing and government policies that restrict income support or access to employment and education. For example, Ghazi, an asylum seeker from the Middle East, said: *“[m]ost landlords they don’t like refugee”*, and Banou and Daina -both refugees from Africa with large families - also indicated housing was a location of covert discrimination from real estate professionals. For instance, Banou said: “*there is a sneaky way of not giving me the house I apply for”.* Naeva’s daughter (interpreting) also referred to her mother not being successful in securing a house because of intersecting discrimination based on race/ethnicity, religion and gender: *“because most of the people, once they see especially what she’s wearing* [a headscarf] *they say ‘this kind of Muslim*’”.

Others referred to discriminatory government policies, specifically restrictions on temporary visa holders. For instance, Middle Eastern asylum seeker Fabienne (female) said: *“I don’t know why the government is doing this because, you know, like those refugees who are on permanent visa, you know, they can go to TAFE* [technical college] *and take some courses. For us -- you know, there is no facilities for us”.* Anahita (Middle East, female, TV) also highlighted the greater difficulty for those on temporary visas in accessing employment: *“They* [potential employers] *ask me what kind of visa I’m on, and when I say bridging visa, they say to me ‘have a good day, bye’”*), and health services: *“because of the type of visa, they say no… to give you that kind of services.”* Shabir similarly noted restrictions based on his temporary visa status:

*Whatever I need help, whenever I’m going, firstly they’re going to ask me my visa condition […] If I’m going to say ‘no, I’ve got a temporary visa or bridging visa’. ‘Sorry, we are not allowed to help’ which is …it’s very sad for us. It’s very sad* (Middle East, TV).

#### Responses and health

Participants reported affective, cognitive and behavioural responses to these experiences of discrimination, all with potential impacts on health, with response type spanning incident types. Participants also responded to individual incidents in more than one way, and used different responses depending on the situation.

##### Affective responses

Key affective responses to experiences of discrimination were anger, fear, frustration and hopelessness, sadness, and shame (a number of which were evident in the accounts above), which all had direct effects on health. For example, the experience of violent assault recounted above by Naweed - as well as the vicarious impact of the discrimination leveled at his wife - left him angry and with lasting impacts on his health:

*I get angry […] now every time when I remember that time the stress comes to me for one hour. Especially if I go to the bed, if this memory comes to my brain I will not go to sleep; I will lose my sleep.*

Fear was also highlighted by participants, particularly where discrimination occurred in their own neighbourhoods – “*yeah they made me fear […] I was always in fear”* (Banou, Africa, female, PV)*.* Likewise, Rachel’s (SE Asia, PV) neighbour’s threatening behaviour was her family’s first encounter with an Australian, which caused them to fear all Australians, and, “*lose confidence*.”

A sense of frustration and hopelessness was particularly expressed by those on temporary visas, who felt that taking any action would likely be unsuccessful and may negatively affect their visa status. For example, Anahita (Middle East, TV) discussed her lack of rights in Australia, which affected her mental health:

*If I knew we would have such a kind of life, and … be treated like this, never ever. I’d prefer to be killed there, just… look at me. I’m like a moving body… There’s no soul, there’s no life in me.*

Participants also discussed more general emotional reactions. For example, Janan (Middle East, TV*)* - in response to being treated differently due to his Afghani origin - said “*I get sad sometimes - why are people thinking like that because I’m different?”*, and Robel (Africa, PV) described his emotional reactions to being ignored and treated differently by his peers at university: “*I’m emotional because - [at the time] and it takes around two, three days to just [gone], yeah, during that time my mental health is not in good condition.”* Vicarious experiences also led to affective responses. For example, Banou (Africa, PV) described feeling *“upset”* when hearing about her children’s experiences of discrimination at school.

Feelings of shame were also evident in participants’ accounts, particularly amongst female participants through the use of the phrase or phrases similar to “*I don’t want to talk about it*” and *“Don’t want to say”*. For example, asylum seeker Eli, noted: *“On the bus, yes. I was very ashamed. I don’t want to talk about it*” (Middle East, female). These affective responses highlight the importance of considering under-reporting of experiences of discrimination and also the potential for internalised discrimination.

##### Cognitive responses

Participants also used cognitive strategies in their responses, particularly through ignoring discrimination or exercising ‘patience’. For example, Robel (Africa, PV), in relation to his multiple experiences of discrimination and their impact said: “*at the time it really hurts but get over it in 2-3 days […], I’m patient”.* Likewise, Eskandar (Middle East, TV) describes using patience as a strategy, although this also resulted in reducing his level of openness to others: “*I have a lot of patience so I just put my head down and close my heart”*. Similarly, Bijan (Middle East, TV) explicitly linked discrimination with her health, but responded by ignoring it: “*if I cared more, yes, this effect on my health but I ignore it. I try ignore it”*.

Other participants also downplayed any ill effects. For example: “*I ignore most of the - if I try this and if it doesn’t work I just like give up, I don’t push; that’s my nature. It didn’t affect me anyway”* (Esron, Africa, male, PV), and *“I tried to handle the situation and not to think about it and process that and not to put any bad effects on me”* (Payam, Middle East, male, TV). However, Payam also goes on to say in relation to the impact of discrimination on health: *“[i]f I say it doesn’t impact anything I’m lying, to be honest with you*”, highlighting the potential limitations of this strategy. Interestingly, for Payam (and others) the cognitive response of ignoring and downplaying discrimination was used in light of constraints in confronting the situation or person. For Payam this was framed as an issue of language:

“*I tried to ignore them because the first problem is my language. My language is not that good to try to discuss and to talk to them and, talk them out of this kind of thinking which they have.”*

A further cognitive response strategy employed by a number of participants was framing discrimination as not unique to Australia and therefore that their new country was not a source of particular harm to their health. For example: Patrick (Africa, male, PV) says “*I think discrimination is everywhere -people that we are born in the same country, within the tribes there will be some discrimination”;* Adahsir (Middle East, male, PV) stressed *“because everywhere, every country you can see it”;* and Solomon said *“On average I tell you, people are fantastic. I have my very positive experience with Australians”* (Africa, male, PV)*.* Samson (Africa, male, PV) also framed discrimination as universal, and described ‘passing’ the phase whereby it affected him, reflecting the other cognitive strategy of minimising harm:

*In my path, personally, I didn’t affect with any discrimination because I was also in Europe so I passed that phase […] I know the place where I am living is originally or is - everyone is immigrant so I know that on my mind so I don’t feel any discrimination in any place.*

For some this strategy was also used in tandem with recognising negative health impacts of discrimination. For example, Naweed, who described significant health effects stemming from the physical assault also stressed his experience of discrimination as unusual: *“I had only one which was difficult for us but not [more]. I could say maybe 95 percent of people in my opinion were good with their good behaviour.”*

##### Behavioural responses

Key behavioural strategies were undertaken to reduce the chance of experiencing discrimination (removing visible signs of religion, not going out, moving house or changing name) and in a minority of cases to confront the perpetrators/situation.

For example, Vashti (Middle East, female, TV) discussed both affective (sadness) and behavioural (removing scarf) responses to incivility:

*I [hear] some voice in the [car] and they’re bullying me […] then I take off my scarf. Maybe this country doesn’t like Muslims […] They say ‘you are Muslim. Why you are coming to this country?’ and I said ‘I’m not Muslim. I don’t have any religion’ […] Make me sad.*

Vashti also reported using cognitive strategies such as ignoring people in response to experiences of incivility, *“Often on the bus and some public places we hear from people who are rude and they are talking about us but we don’t speak at all.”*

A number of participants reported moderating their movements/activities significantly in light of discrimination experiences. For example, Kiarna said: “*I’m happy to lock the door and avoid going outside after it is dark”* (Middle East, female, TV) and Eskander (Middle East, TV) similarly restricted his movements: “*past 9 o’ clock I can’t go out, I’m scared. Home before dark, can’t go out after dark”.*

Other behavioural strategies were noted by participants for example, Shabir (Middle East, TV) attempted to change his name to a “Western” sounding name to avoid discrimination, though his visa status prevented this: *“my name is - is kind of Muslim but I decide to change my name but ...They said ‘you need to be Australian citizen’. ...so still I am [stuck] with my name”.*

Several participants, such as Rachel above and Solomon: (“*I saw the property owner was not respectful of my background”*, male, Africa, PV) also reported moving from their house or neighbourhood due to the discrimination they experienced.

Only four participants described attempts to take direct action. Two addressed the perpetrators and distanced themselves from the ‘subject’ of the discrimination – in each case Islam. For example, Farhad, a refugee from the Middle East who is Christian, described his response to anti-Muslim remarks two men were making towards him when he was fishing, after ignoring it was unsuccessful:

*When I heard I thought it’s only once and then I ignored. They are continuing so l pack all of my stuff and left that place, but before leaving I told them ‘sorry, I’m not Muslim, I’m Christian’.*

Naweed reported the physical assault and the discrimination targeted at his wife, described above, to the police but received an inadequate response. After several attempts to follow this up his wife asked him to drop it because she was worried about the impact it was having on his health. Banou had complained to the local housing authority about the racial abuse she had experienced from another tenant, but nothing had been done.

#### Underreporting

Some participants described incidents of discrimination in the interview but had not indicated this in the survey. This may reflect the limitations of survey methods in collating sensitive information or differences in question wording and may also reflect the cognitive response of minimising the impact of discrimination noted above. However, there was also evidence of some reluctance to discuss and name discrimination with ‘Australian’ interviewers – both so as not to appear ‘ungrateful’ but also out of fear of potential impacts on visa determination. For instance, Anahita (Middle East, female, TV) said:

*When I was about to come to this interview, I told my friends and my friends said not to say anything, because definitely it would have effect on your visa, and you know, you shouldn’t say these things against the government, because if you [do] definitely you will lose your visa*.

Reports of discrimination as a ‘minority’ of experiences (above) may also reflect a desire to make the interviewer more comfortable. For example, Farhad (Middle East, male, PV) was mindful of not upsetting the interviewer, saying: “s*o you are Australian and I’m not going to make you sad but I think -- so [I’ll say] that they do not make any discrimination, but 50 percent are doing that.”*

These accounts underscore the potential for underreporting, as well as perceived constraints in taking more direct action.

## Discussion

This paper highlights the broad and extensive experiences of and responses to discrimination reported by refugees and asylum seekers in Australia, and associated impacts on health. Discrimination featured in the resettlement experiences of over 1 in 5 survey respondents and over half of the interviewees – although this is likely an underestimate - and there was also evidence of vicarious discrimination. These experiences occurred in a range of settings and included incivility, threats and physical assault as well as unequal access to resources, and involved intersecting categories of visa or immigration status, race/ethnicity, culture, religion and gender. Participants reported clear negative impacts on health and responses to discriminatory experiences spanned affective, cognitive and behavioural dimensions. Despite significant acts of agency and resistance in participants’ accounts, structural factors - particularly for asylum seekers - constrained responses, and the ‘cost’ required to mitigate the impacts of discrimination was also evident. This, coupled with the links to negative health impacts, highlights discrimination as a critical resettlement issue for refugees and asylum seekers.

The high levels and wide-ranging experiences of discrimination reported are noteworthy. The survey figure was comparable to the 20% found in the general Australian population Scanlon Foundation survey in 2016, which used the same survey question [68], and interview participants reported higher rates. Other studies have found varied rates of discrimination. For example, Noh et al. using a single measure found 26% of his sample of refugees from Southeast Asia resettled in Canada reported discrimination on the basis of ‘race’ [69] and Willis and Nkwocha also using a single item found 53% of Sudanese refugees in the USA reported experiencing racism [70]. Hadley and Patil using a multi-item measure found that 52% of their sample of refugees from Africa and Eastern Europe resettled in the USA reported experiencing racism [71]. In Australia Fozdar and Torezani found that 47% of their sample of refugees from the former Yugoslavia, the Middle East and Africa reported being discriminated against in the job market [39]. These variations in rates may relate to the measures used, the nature of the sample and the resettlement context. Our study had a lower rate than most of these studies. This may relate to the survey item used. We also note the evidence of underreporting in this study, potentially due to issues of shame, social desirability, a ‘politeness imperative’ or perceived expectation to engage discourses of gratitude in describing resettlement experiences [39, 72]. In addition, the fear of potentially negative consequences for visa determination of identifying discrimination may also have contributed to underreporting - which may have been particularly strong for the asylum seekers in our study (most other studies have only examined those with confirmed refugee status). Participants also observed the sometimes covert nature of discrimination, which could make it harder to ‘name’, and which may also lead to underreporting. We also found evidence of vicarious discrimination, which has been shown to adversely affect health [22–24].

We found higher rates of discrimination by those from the Middle East and Africa – mirroring other studies that have identified differences by country of origin (e.g. [39, 71], and also found higher rates of discrimination for those on temporary visas. The qualitative data highlighted that experiences of discrimination occurred at the intersection of visa status and a number of other social categories - particularly race/ethnicity for participants from Africa; gender, race/ethnicity, and religion for Muslim women; and race/ethnicity, and religion for asylum seekers from the Middle East. As such, discrimination was often specifically targeted at refugees and asylum seekers due to their migration pathway, over and above their race/ethnicity, culture or religion – which has been found elsewhere [45, 46]. The study also highlights the complex identities found within the social categories of ‘refugee’ and ‘asylum seeker’, the compounding disadvantage faced by this group, and the value of considering how multiple identities can interact to intensify discrimination [28–30].

In relation to these complex identities, the higher rates of discrimination for those with no religion (including religious discrimination) was surprising but may relate to the multifaceted way in which identity characteristics of race/ethnicity, culture and religion coalesce. As noted, the majority of those who reported no-religion were asylum seekers from the Middle East, and ‘Muslim’ identities may have been ascribed to people due to their Middle Eastern appearance or ‘religious’ dress (e.g. hijab). This highlights the potential impact of broader racialising of religion and islamophobia [32, 33, 73]. Attempts by participants to actively avoid racism by specifically noting that they were not Muslim, or by removing visible markers associated with Islam - highlighted an awareness of Islamophobia in Australia. Similarly noteworthy was the relative absence of discrimination for those from SE Asia, which may relate to the younger age, permanent visa status, and more recent arrival of this group. Overall, then, the study highlights that consideration of intersecting identities is crucial to understanding the discrimination experiences of refugees and asylum seekers, and the varied impacts that these experiences have [74].

Experiences of discrimination were associated with negative impacts on health, supporting a growing body of research in this area [21, 40, 43, 45–56]. Interestingly physical health was not significantly worse for those who had experienced discrimination, and qualitative descriptions of impacts on physical health were rare, focusing largely on sleep disturbance (in addition to the direct impact of physical assault). It may be that people were more conscious of impacts on mental health or that pathways to physical health effects are more complex.

The broad range of settings in which people experienced discrimination (e.g. education, housing, neighbourhoods) are all important elements of successful resettlement and integration [75] and represent key social determinants of health [57]. Thus discrimination in relation to these elements and behavioural responses evidenced in this research (e.g., restricting movement outside the home) are likely to have indirect effects on health [76]. The survey also found lower levels of trust, control, hope and belonging amongst those who had experienced discrimination and similar links were evident in the interviews. Each of these elements has an impact on integration and a sense of safety, which is of particular importance for health for refugees and asylum seekers given the likelihood of previous experiences of trauma and threats to personal security.

Participants’ responses to discrimination spanned affective, cognitive, and behavioral elements, often in combination, and reflect some of the key responses to discrimination of other groups in Australia, for example Aboriginal Australians, [59, 77]. In relation to types of incidents and types of responses no consistent pattern could be found to explain particular responses. One incident could lead to multiple types of responses for some, and for others responses depended on the incident and/or context. However, what was clear was that those on temporary visas felt that more direct responses were not available to them for fear of an impact on visa determination and low likelihood of being listened to. Indeed, across the participant sample there were few examples of confronting the perpetrator, and no reports of taking action leading to a positive outcome.

In general, the responses evident in this research could be seen as ‘passive’, which have been found to be less health protective than responses such as confronting the perpetrator [9, 11, 13, 58]. However, in participants’ accounts there were also clear examples of agency, with some participants framing their responses as a ‘choice’ (e.g. ignoring an incident or choosing not to let it affect them), which has been shown to be protective. Overall, the health protective value of particular response types is likely dependent on context as well as the population group.

These findings need to be considered in the context of pre-migration and post-migration factors. Firstly, pre-migratory trauma may make experiences of discrimination in Australia seem minor in comparison [39] but could also further sensitise people to discriminatory actions, particularly those accompanied by a sense of threat. Secondly, pre-migratory experiences (and post-migratory, particularly for those who had experienced Australian immigration detention) may also make people distrustful of authorities and therefore less likely to make a formal complaint. Thirdly, and relatedly, for asylum seekers in particular, concerns about visa status in Australia may make them reluctant to complain, and the use of a ‘character test’ in visa determination processes in Australia may exacerbate this fear. Likewise, discriminatory government policies and negative political discourse about immigration, and in particular about asylum seekers, has contributed to creating environments where discrimination can flourish [78–80] and authorities may be seen as complicit in this. Fourthly, the ‘politeness principle’ and discourses of gratitude [39] may lead to underreporting and also prevent people from making a discrimination complaint (or reporting it in research). Finally, most refugees and asylum seekers come from collectivist cultures where, as Noh et al. (1999) suggest, there may be less emphasis on ‘taking action’ in the face of discrimination and more emphasis on cooperation and avoidance of conflict. Within such cultural settings, ‘passive’ responses such as forbearance may have greater ‘cultural congruency’ and be more effective coping strategies.

It was also clear from the interviews that the relative ‘protective’ value of various strategies is likely to be situationally determined and there was substantial ‘work’ and a ‘cost’ associated with coping involved in framing responses, that can also undermine health [11, 58, 59]. Clearly the goal is for discrimination not to be experienced and such work not to be required. Much of the research on responses to discrimination has been on individual responses rather than more structural responses such as anti-racism legislation or workplace policies – the utility of these higher level responses in the case of refugees and asylum seekers is an important area of policy action and further research [40].

In this way there are a range of actions that could be considered to address discrimination and its harmful impacts on refugees and asylum seekers, which should be further developed in collaboration with refugee and asylum seeker communities. Firstly, cessation of inflammatory, demonising and discriminatory language in political (and media) debates about immigration is essential [81–85]. Secondly, discriminatory government policies that restrict access to essential services for some groups of refugees and asylum seekers need to be reviewed. Thirdly, specific initiatives to address discrimination affecting refugees and asylum seekers are required and broader anti-discrimination policies and programs should include examples of people from refugee and asylum seeker backgrounds and highlight the impact of intersecting forms of discrimination and the potentially compounding effects [83, 84, 86]. Fourthly, education for refugees and asylum seekers about anti-discrimination legislation and policy in Australia may assist in helping new arrivals understand their rights and protections. Fifthly, there needs to be outreach to refugee and asylum seeker communities by discrimination complaints authorities (adequately resourced to do so) to facilitate the lodgment of complaints by those who have experienced discrimination. The Victorian Equal Opportunity and Human Rights Commission is currently trialing a community reporting tool to facilitate racism complaints that may be of value [87]. Complainants in all schemes should be given protection from government ‘character tests’ - for example, if a workplace discrimination complaint is made by someone without work-rights. Sixthly, community development programs, particularly those at a neighbourhood level given this as a prominent site for discrimination, that seek to build social cohesion are very important and require resourcing. Finally, adequate funding for appropriate counselling and support services around issues of discrimination is crucial – the compounding impact of discrimination for those who have fled persecution warrants specialist support expertise.

There were some limitations to the study. The survey was cross-sectional and used a convenience sample and we are unable to assess a ‘refusal’ rate given the snowball and other sampling employed. The findings therefore cannot be generalised to the broader population. However, through a mix of sampling we were able to reach people who generally don’t participate in research (e.g. due to literacy issues) and there are also bias risks in probability sampling [48]. The sample size prohibited a more nuanced quantitative analysis of intersecting characteristics. In the qualitative analysis varied demographic characteristics across the different cultural/ethnic/racial groups made a consistent analysis of the intersections more difficult and warrants further research. We also note that the survey question in relation to discrimination was a single item and focused on skin colour, ethnic origin and religion and did not specifically ask about other factors such as gender nor visa (although open-ended responses did reflect these elements). The interview theme guide allowed for a broader discussion of discrimination and its potential origins. While we piloted our measures with refugee and asylum seeker communities and the project was guided by a working party of people from refugee and asylum seeking backgrounds and a Steering Group of service providers working with refugees, we note the issue of cultural appropriateness of survey measures, including the SF-8, in general and also for refugees in particular [88–90]. There is a difficult balance between using measures specifically developed for populations and more general measures that facilitate comparisons with the general population [64, 91, 92].

## Conclusion

Discrimination harms resettlement and integration for refugees and asylum seekers as well as health, and for those fleeing trauma and persecution it can be particularly detrimental. This study highlights the nature, extent, responses and consequences of discrimination experienced by refugees and asylum seekers resettled in Australia. There is a clear moral imperative to address discrimination in resettlement countries if they are to fulfill their obligations to provide a ‘safe haven’ for those who seek protection.

## Supplementary information

**Additional file 1.** 'Belonging Begins at Home' Survey.

**Additional file 2.** 'Belonging Begins at Home' semi-structured interview guide.

## Data Availability

Due to ethical concerns, supporting data cannot be made openly available. Please contact the author for further information about the data and conditions for access.

## Abbreviations

MCS: Mental health composite score
PCS: Physical health composite score
PV: Permanent Visa
SE Asia: Southeast Asia
SF-8: Short Form 8 (health measure)
TV: Temporary Visa
UNHCR: United Nations High Commissioner for Refugees
