# Supplementary material for: Discrimination: a health hazard for people from refugee and asylum-seeking backgrounds resettled in Australia
Source: BMC Public Health. 2020 Jan 28;20:108. doi: 10.1186/s12889-019-8068-3 (PMC6986068; doi:10.1186/s12889-019-8068-3)
Supplement: Supplementary file 1 — Additional file 1. 'Belonging Begins at Home' Survey. [file 12889_2019_8068_MOESM1_ESM.docx]

**Additional file 1: ‘Belonging Begins at Home’ Survey**

**
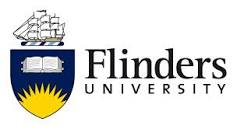
Housing and Wellbeing for**

**Refugees and Asylum Seekers in South Australia**

This survey is for people aged **18 years and over** with **refugee and asylum seeker backgrounds** who are currently living in South Australia, and have lived in Australia for **less than 7 years.**

We are interested in your experiences of housing in Australia

and your health and wellbeing.

**Please read these instructions before answering the questions:**

- We think this will take you around 30 minutes to complete and you can receive a $20 voucher.
- We do not need your name on the survey, and your answers will be confidential.
- Please only complete this survey once.
- The word “housing” means any sort of accommodation that you live in, including a house, a flat or an apartment.
- There are no right or wrong answers. For most questions, please tick only one box (the one that most applies to you). Some questions have the option to tick more than one box, and this will be part of the question instructions.
- When you have finished filling in the survey, you can put it in the large envelope and either give it to the researchers or put it in the post. **You do not need to put a stamp on it.** Instructions for the $20 shopping voucher are at the end of the survey.
- If you need any help to complete the survey please speak to the researchers, or service provider/community organisation staff or volunteers. You can also contact Clemmi on 7221 8478 or clemence.due@flinders.edu.au

If you would prefer to complete this survey online, you can go to <https://www.surveymonkey.com/r/refugeehousing>.

**Thank you**

This survey is part of the ‘*Belonging begins at home: Promoting social inclusion and wellbeing for people from refugee backgrounds’* project*.* This project will investigate the impact of housing on health, wellbeing and social inclusion.

Sharing your experiences will help to show how important housing might be for the health of refugees in Australia. The survey is voluntary and you do not have to answer the questions, or even start the survey. If you say no, your life in Australia will not be affected in any way.

If you or anyone else you know would like a copy of the findings of the survey, tick ‘yes’ on the form at the end of the survey. We will send you a copy of the findings.

**Investigators:**

Assoc Prof Anna Ziersch

Southgate Institute for Health, Society and Equity

Flinders University

Ph: 08 7221 8484

Assoc Prof Kathy Arthurson

Southgate Institute for Health, Society and Equity

Flinders University

Ph: 08 7221 8416

Dr Clemence Due

Southgate Institute for Health, Society and Equity

Flinders University

Ph: 08 7221 8478

Rachel Sullivan

Southgate Institute for Health, Society and Equity

Flinders University

We don’t think that the questions will make you feel uncomfortable or upset. If you do feel upset, you can call Lifeline on 13 11 14. If you need an interpreter, you can call the Translating and Interpreting Service on 13 14 50 and ask them to call Lifeline for you on 13 11 14. You will also receive a copy of a sheet with some other organisations who can help if you feel upset. They are there to help you.

*This research project has been approved by the Flinders University Social and Behavioural Research Ethics Committee (Project number 6273). For more information regarding ethical approval of the project the Executive Officer of the Committee can be contacted by telephone on 8201 3116, by fax on 8201 2035 or by email* [*human.researchethics@flinders.edu.au*](mailto:human.researchethics@flinders.edu.au)

**Section 1: About You**

1. Please tell us your **age**:

| ❑ | ❑ | ❑ | ❑ | ❑ | ❑ | ❑ | ❑ | ❑ |
| --- | --- | --- | --- | --- | --- | --- | --- | --- |
| 18 to  19 | 20 to 29 | 30 to 39 | 40 to 49 | 50 to 59 | 60 to 69 | 70 to 79 | 80 to 89 | 90 or over |

1. Please tell us your **gender**:

| ❑ | ❑ |
| --- | --- |
| Male | Female |

1. What country were you **born** **in**? _______________________________________________
2. What is your **ethnic/cultural background?** __________________________________
3. What is your **religion?**

| ❑ | Baha’i | ❑ | Islam |
| --- | --- | --- | --- |
| ❑ | Buddhism | ❑ | Judaism |
| ❑ | Christianity | ❑ | I don’t have a religion |
| ❑ | Hinduism | ❑ | I don't want to say |
| ❑ | Other (please write) __________________________________________________ | | |

1. What **languages** do you speak? _________________________________________________
2. How long have you been **living in the community in Australia**? *(Please exclude any time spent in detention centres)*

| ❑ | ❑ | ❑ | ❑ | ❑ | ❑ | ❑ | ❑ | ❑ | ❑ |
| --- | --- | --- | --- | --- | --- | --- | --- | --- | --- |
| Less than 1 month | 1 to 6 months | 7 months to 1 year | 1 year | 2 years | 3 years | 4 years | 5 years | 6 years | 7 years |

1. What is the **highest level of education** that you have completed anywhere?

| ❑ | No formal schooling | ❑ | Trade qualification |
| --- | --- | --- | --- |
| ❑ | Primary school | ❑ | TAFE/technical qualification |
| ❑ | Secondary school (high school) | ❑ | University degree or diploma |
| ❑ | Other (please write) __________________________________________________ | | |

1. What is your **current occupation** in Australia?_______________________________
2. What was your **occupation before you came to Australia**? ________________

______________________________________________________________________________________

1. How **well do you**:

|  | Very well | Well | Not well | Not at all |
| --- | --- | --- | --- | --- |
| Understand spoken English | ❑ | ❑ | ❑ | ❑ |
| Speak English | ❑ | ❑ | ❑ | ❑ |
| Read English | ❑ | ❑ | ❑ | ❑ |
| Write English | ❑ | ❑ | ❑ | ❑ |

1. Do you **have children who are under 18 years old**?

| ❑ | ❑ |
| --- | --- |
| Yes | No |

🡺 If yes, how many **live with you** in Australia? ______________________________

1. Which of the following **visas** did you have **when you arrived** in Australia?

| ❑ | Permanent protection visa  🡺Which type ____________ | ❑ | Temporary protection visa  🡺Which type ________________ |
| --- | --- | --- | --- |
| ❑ | Other  🡺 Which type ____________ | ❑ | No visa |

1. Which of the following **visas** do you **currently have**?

| ❑ | Permanent protection visa/Permanent Resident  🡺Which type _______________ | ❑ | Temporary protection visa  🡺Which type _______________ |
| --- | --- | --- | --- |
| ❑ | Bridging Visa  🡺 Which type _______________ | ❑ | Other  🡺 Which type _______________ |
| ❑ | No visa | ❑ | I am an Australian citizen |
|  |  |  |  |

1. Which of the following **describes your current situation**?
    **Please tick all that apply to you**

| ❑ | Permanent/ongoing work | ❑ | Retired |
| --- | --- | --- | --- |
| ❑ | Fixed-term contract | ❑ | Unemployed |
| ❑ | Casual work | ❑ | Parenting/home duties |
| ❑ | Student/studying | ❑ | Not working because of a disability |
| ❑ | Self employed | ❑ | I don’t have work rights |
| ❑ | Other ___________________________________________________________________________________ | | |

1. Do you have **access to the internet**? **Please tick all that apply to you**

| ❑ | ❑ | ❑ | ❑ | ❑ |
| --- | --- | --- | --- | --- |
| No | Yes, at work | Yes, at home | Yes, on my mobile phone | Yes, elsewhere (such as public library) |

**Section 2: Your Housing and Neighbourhood**

1. **How many** houses have you lived in since you arrived in Australia? (including your current housing) __________________________________________________
2. What type of housing **looks the most like the one you are currently living in?**

| ❑ | Anglicare housing | ❑ | Community Housing |
| --- | --- | --- | --- |
| ❑ | Private rental housing | ❑ | Housing Trust |
| ❑ | Housing that I or my spouse own/pay a mortgage on | ❑ | Temporarily living with family/friends/relatives |
| ❑ | Hotel/motel/caravan | ❑ | Shelter/supported housing |
| ❑ | Boarding house/hostel | ❑ | Homeless/no housing |
| ❑ | Other ___________________________________________________________________________________ | | |

1. Who **lives** in your **current housing** with you? **Please tick all that apply to you**

| ❑ | Live alone | ❑ | Live with children |
| --- | --- | --- | --- |
| ❑ | Live with husband/wife/partner | ❑ | Live with other family/relatives (such as parents, uncles, cousins) |
| ❑ | Live with husband/wife/partner and children | ❑ | Live with friends |
| ❑ | Other ___________________________________________________________________________________ | | |

1. How **long** have you lived in your current housing? _______________________________

| ❑ | ❑ | ❑ | ❑ | ❑ | ❑ | ❑ | ❑ | ❑ | ❑ | ❑ |
| --- | --- | --- | --- | --- | --- | --- | --- | --- | --- | --- |
| Less than 1 month | 1 to 2 months | 3 to 6 months | 7 to 12 months | 1 year | 2 years | 3 years | 4 years | 5 years | 6 years | 7 years |

1. How many people live with you (**including yourself**)? __________________________

🡺 How many of those people are **under 18 years old**? ____________________

1. How many **bedrooms** are there in your current housing?

| ❑ | ❑ | | ❑ | | ❑ | ❑ | ❑ | ❑ |
| --- | --- | --- | --- | --- | --- | --- | --- | --- |
| 0 | | 1 | | 2 | 3 | 4 | 5 | More than 5 |

1. Overall, how do you feel about your **current housing**?

| **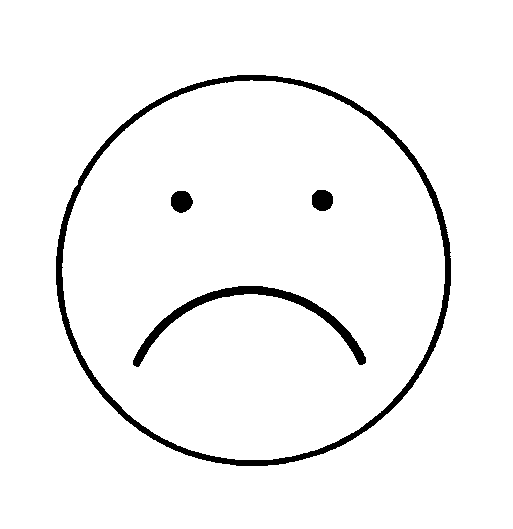** | **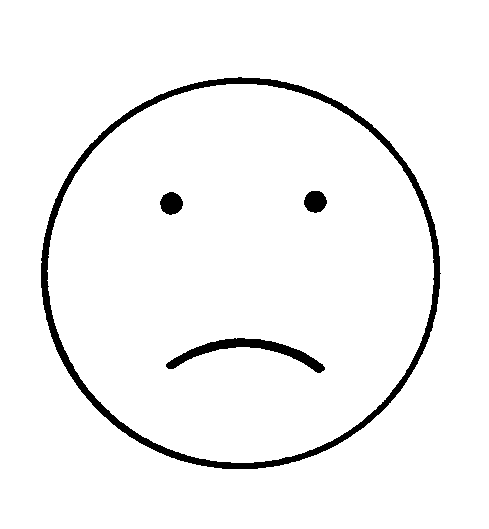** | **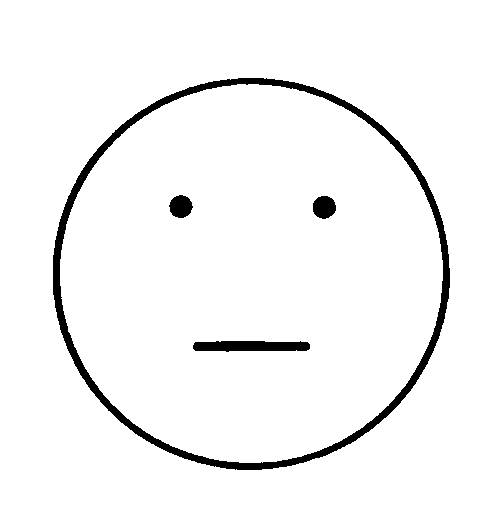** | **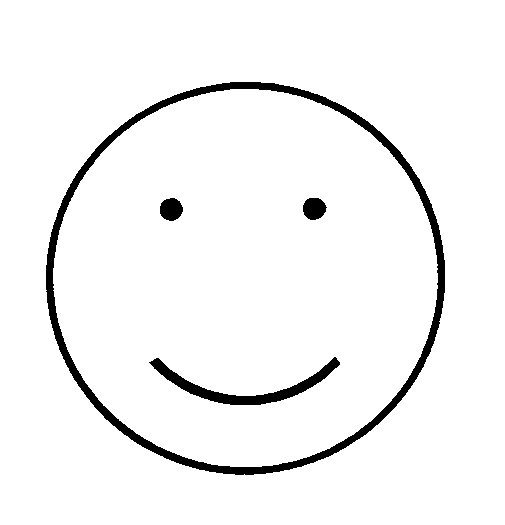** | **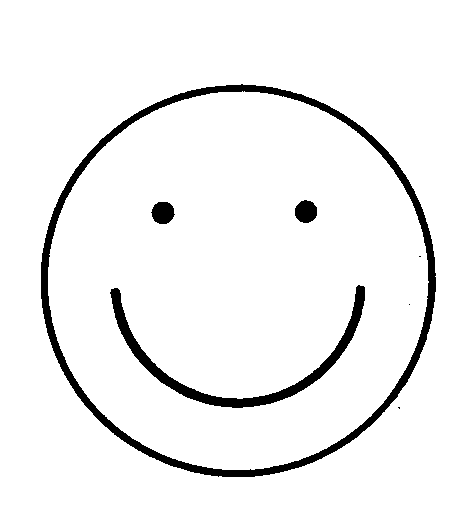** |
| --- | --- | --- | --- | --- |
| ❑  Very unhappy | ❑ | ❑ | ❑ | ❑  Very happy |

🡺 What is the **main** reason for this? **__________________________________________**

1. How did you **find your current housing**? **Please tick all that apply to you**

| ❑ | I found it on my own | ❑ | Through a real estate agent |
| --- | --- | --- | --- |
| ❑ | Through a government or settlement case worker | ❑ | Through friends or family |
| ❑ | Another service provider helped (please tell us who) ______________________________________ | ❑ | Other __________________________________  _________________________________________ |

1. Have you had any of the **problems below** with the **housing** that you **currently live in?** **Please tick all that apply to you**

| ❑ | Getting to open inspections | ❑ | Heating and cooling |
| --- | --- | --- | --- |
| ❑ | Applying for public housing | ❑ | The rent is too expensive |
| ❑ | Securing housing due to large family | ❑ | Getting things fixed |
| ❑ | No referees/rental history | ❑ | Difficulty with periodic inspections |
| ❑ | Lack of affordable housing in the area I want to live in | ❑ | Understanding my rights and responsibilities as a tenant |
| ❑ | Getting a mortgage | ❑ | Not enough bedrooms |
| ❑ | Problems with interpreters | ❑ | Not enough living areas |
| ❑ | Communicating in English | ❑ | Not enough bathrooms |
| ❑ | Problems with real estate agents or landlords | ❑ | Looking after the housing and garden |
| ❑ | Housing too crowded | ❑ | Difficulty with neighbours |
| ❑ | Housing not in good condition | ❑ | Discrimination |
| ❑ | Housing not safe | ❑ | Getting bond returned |
| ❑ | Other | ❑ | I haven’t had any problems |
|  | ________________________________________________________________________________________  _________________________________________________________________________________ | | |

1. Have you needed **help with any of the problems** with **your current housing that you ticked** in the previous question?

| ❑ | ❑ | ❑ | ❑ |
| --- | --- | --- | --- |
| Yes, a small amount of help | Yes, a lot of help | No | I haven’t had any problems |

🡺 If you needed help, please tell us **who** helped you and **how**?

1. What **suburb** do you live in?________________________________________________________
2. How do you feel about your **current neighbourhood**?

| **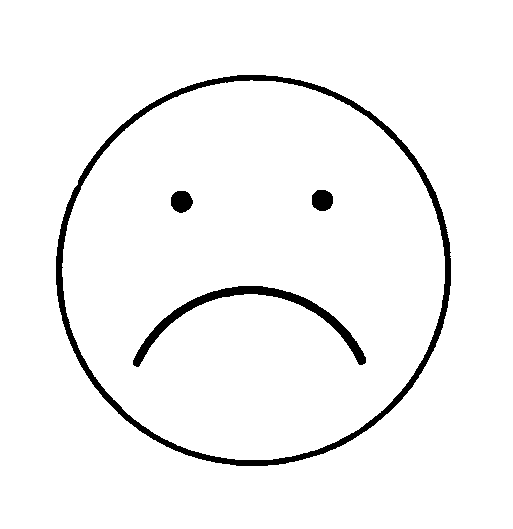** | **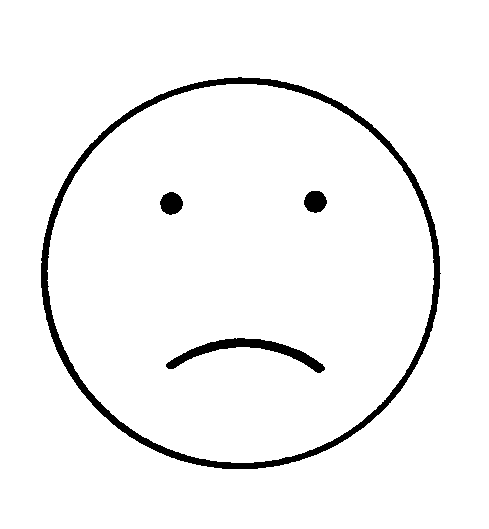** | **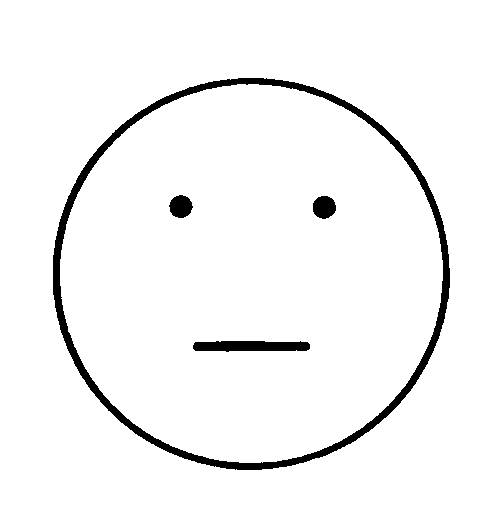** | **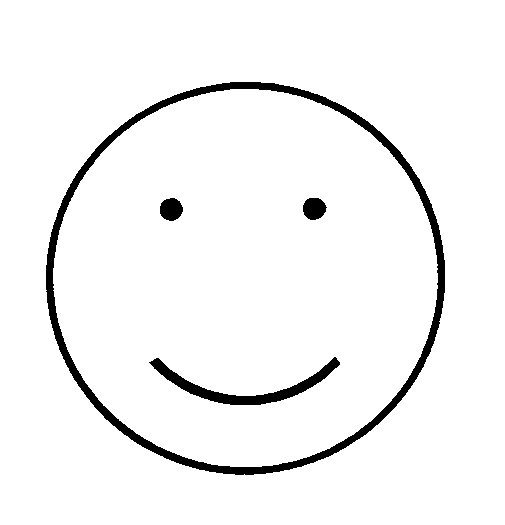** | **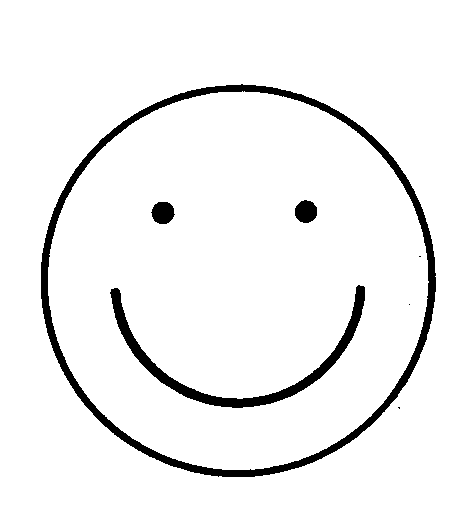** |
| --- | --- | --- | --- | --- |
| ❑  Very unhappy | ❑ | ❑ | ❑ | ❑  Very happy |

🡺 What is the **main** reason for this? **____________________________________________**

**_______________________________________________________________________________________**

1. Have you had any of the **problems below in your current neighbourhood?**

**Please tick all that apply to you**

| ❑ | The neighbourhood is not safe | ❑ | Too far from schools/childcare |
| --- | --- | --- | --- |
| ❑ | The neighbourhood is not friendly | ❑ | Too far from public transport |
| ❑ | Too far from family/relatives | ❑ | Too far from work |
| ❑ | Too far from friends | ❑ | Trouble with neighbours |
| ❑ | Too far from place of worship | ❑ | Not enough local services (such as libraries and doctors) |
| ❑ | Too far from shops | ❑ | I haven’t had any problems |
| ❑ | Other ___________________________________________________________________________________ | | |
|  | __________________________________________________________________________________ | | |

1. Are you currently **looking for other housing**?

| ❑ | ❑ |
| --- | --- |
| Yes | No |

1. **In the next 6 to12 months,** what are your **plans** for housing?

| ❑ | Stay in the house I live in | ❑ | Move to Community Housing |
| --- | --- | --- | --- |
| ❑ | Move and rent a house privately | ❑ | Move to Housing Trust |
| ❑ | Move and buy a house | ❑ | Don’t know |
| ❑ | Move and live with family/relatives or friends |  |  |
| ❑ | Other ___________________________________________________________________________________ | | |

1. Do you think that you will **need help to find your next housing**?

| ❑ | ❑ | ❑ | ❑ |
| --- | --- | --- | --- |
| Yes, a small amount of help | Yes, a lot of help | No | Not planning on moving |

1. What is **important to you** when **choosing housing**?

**Please tick all that apply to you**

| ❑ | Affordable rent | ❑ | A garden/yard |
| --- | --- | --- | --- |
| ❑ | Housing you are buying or own | ❑ | In good condition |
| ❑ | Enough bedrooms | ❑ | Housing in which you feel safe |
| ❑ | Enough living areas | ❑ | In a good neighbourhood |
| ❑ | Enough bathrooms |  |  |
| ❑ | Other ___________________________________________________________________________________ | | |

1. What is **important to you** when **choosing a neighbourhood**?

**Please tick all that apply to you**

| ❑ | Feeling safe | ❑ | Close to schools/childcare |
| --- | --- | --- | --- |
| ❑ | Friendly | ❑ | Good public transport |
| ❑ | Close to relatives/family | ❑ | Close to work |
| ❑ | Close to friends | ❑ | Good neighbours |
| ❑ | Close to place of worship | ❑ | Good local services (such as libraries and doctors) |
| ❑ | Close to shops |  |  |
| ❑ | Other ___________________________________________________________________________________ | | |

Have you lived in **more than one house** in Australia?

| ❑ | No 🡺 please skip the green page and go to question 37 |
| --- | --- |
| ❑ | Yes 🡺 please answer the questions on the green page |

**ONLY ANSWER THE QUESTIONS 35 AND 36 ON THIS GREEN SHEET IF YOU HAVE LIVED IN MORE THAN ONE HOUSE IN AUSTRALIA**

*(SKIP TO THE NEXT PAGE IF YOU HAVE ONLY LIVED IN ONE HOUSE)*

1. a): **When you first arrived in Australia** where did you live?

State: __________________ Suburb: _____________________ Town:__________________

b) W**hat housing did you first live in**?

| ❑ | Anglicare housing | ❑ | Private rental housing I found myself | |
| --- | --- | --- | --- | --- |
| ❑ | Housing provided by another service provider 🡺 please tell us who_________________________________ | ❑ | Private rental housing found by a service provider | |
| ❑ | Temporarily lived with family/friends/relatives | ❑ | Housing that I or my spouse owned/paid mortgage on | |
| ❑ | Housing Trust | ❑ | Hotel/motel/caravan | |
| ❑ | Community Housing | ❑ | Shelter/supported housing | |
| ❑ | Boarding house/hostel | ❑ | Homeless/no housing | |
| ❑ | Other 🡺Please tell us __________________________________­­­­­­­­­­­­­­­­­­­­­­­____________________ | | |  |

c) How **long did you live in this housing**?

| ❑ | ❑ | ❑ | ❑ | ❑ |
| --- | --- | --- | --- | --- |
| Less than 1 month | 1 to 2 months | 3 to 6 months | 7 to 12 months | Over 1 year |

d) How **happy were you with this housing**?

| **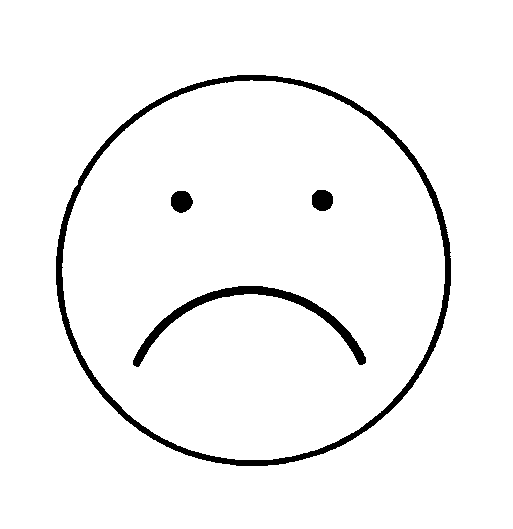** | **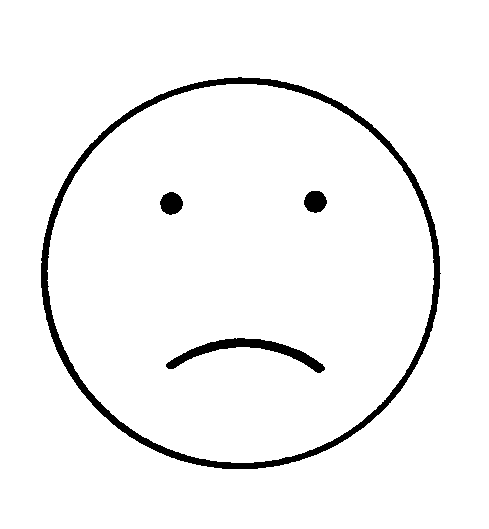** | **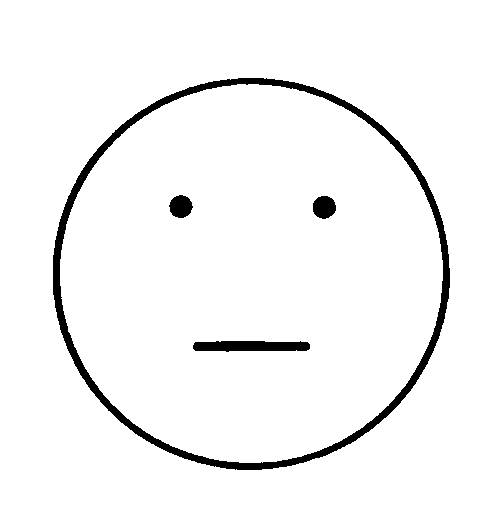** | **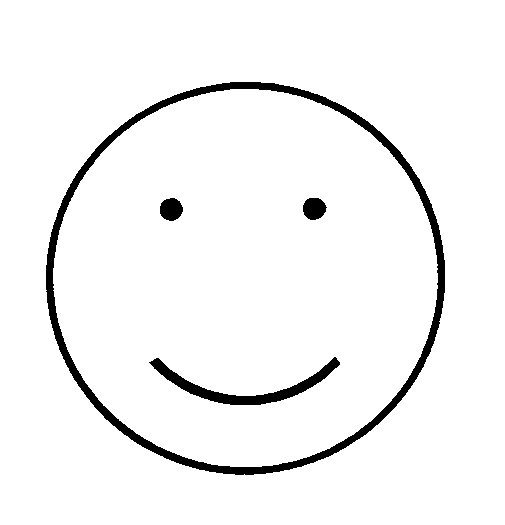** | **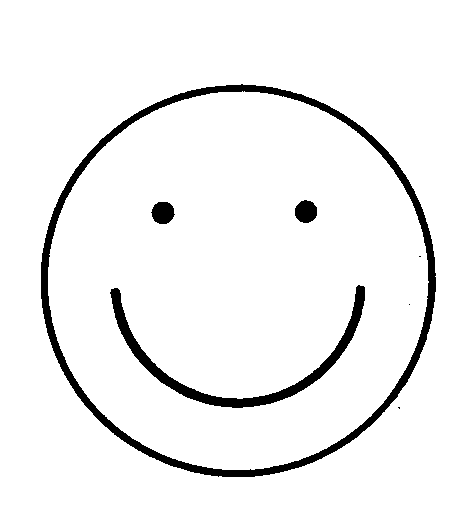** |
| --- | --- | --- | --- | --- |
| ❑  Very unhappy | ❑ | ❑ | ❑ | ❑  Very happy |

🡺What is the **main** reason for this? ______________________________________

e) Why did you **move** from this **housing**? ________________________________________

f) How did you find the **housing you moved to after you this first one**?

**Please tick all that apply to you**

| ❑ | I found it on my own | ❑ | Through a real estate agent |
| --- | --- | --- | --- |
| ❑ | Through a government or settlement case worker | ❑ | Through friends or family |
| ❑ | Another service provider helped (please tell us who) ______________________________________ | ❑ | Other _________________________________  ________________________________________ |

g) Have you **needed help to find other housing since then**?

| ❑ | ❑ | ❑ |
| --- | --- | --- |
| Yes, a small amount | Yes, a lot of help | No, I haven’t needed any help |

🡺 If you needed help, please tell us who helped you and how?

1. Have you had any of the **problems below** in **housing** (apart from your current housing) in Australia?

**Please tick all that apply to you**

| ❑ | Getting to open inspections | ❑ | Heating and cooling |
| --- | --- | --- | --- |
| ❑ | Applying for public housing | ❑ | The rent was too expensive |
| ❑ | Securing housing due to large family | ❑ | Getting things fixed |
| ❑ | No referees/rental history | ❑ | Difficulty with periodic inspections |
| ❑ | Lack of affordable housing in the area I want to live in | ❑ | Understanding my rights and responsibilities as a tenant |
| ❑ | Getting a mortgage | ❑ | Not enough bedrooms |
| ❑ | Problems with interpreters | ❑ | Not enough living areas |
| ❑ | Communicating in English | ❑ | Not enough bathrooms |
| ❑ | Problems with real estate agents or landlords | ❑ | Looking after the housing and garden |
| ❑ | Housing too crowded | ❑ | Difficulty with neighbours |
| ❑ | Housing not in good condition | ❑ | Discrimination |
| ❑ | Housing not safe | ❑ | Getting bond returned |
| ❑ | Other _________________________________ | ❑ | I haven’t had any problems |
|  | __________________________________________ | | |

🡺 What **other suburbs** have you lived in? _______________________________________

🡺 Have you had any of the **problems below in any neighbourhood in Australia** (other than the one you currently live in)**?**

**Please tick all that apply to you**

| ❑ | The neighbourhood was not safe | ❑ | Too far from shops |
| --- | --- | --- | --- |
| ❑ | The neighbourhood was not friendly | ❑ | Too far from schools/childcare |
| ❑ | Too far from family/relatives | ❑ | Too far from public transport |
| ❑ | Too far from friends | ❑ | Too far from work |
| ❑ | Too far from place of worship | ❑ | Trouble with neighbours |
| ❑ | Not enough local services (such as libraries and doctors) | ❑ | Other________________________________  _______________________________________ |
| ❑ | I haven’t had any problems | | |

**Section 3: Your Activities and Social Engagement**

1. Please tell us about **your social connections with people**. For example, who do you spend time with and in what way?
2. Overall, how **often do you socialise?**

| ❑ | ❑ | ❑ | ❑ | ❑ |
| --- | --- | --- | --- | --- |
| Every day | Several times a week | Once a week | Several times a month | Once a month or less |

1. How happy are you with your **social connections** to the people in the **neighbourhood where you live**?

| **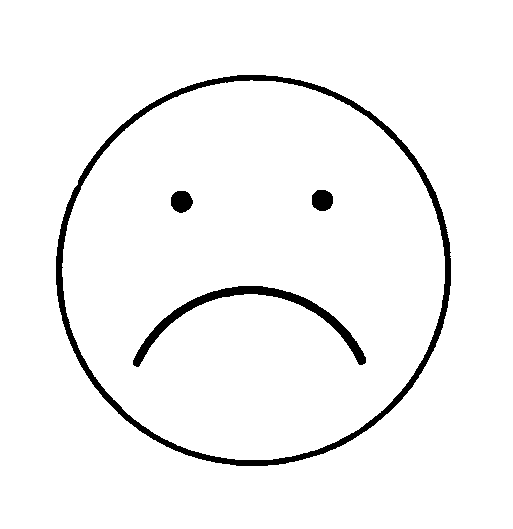** | **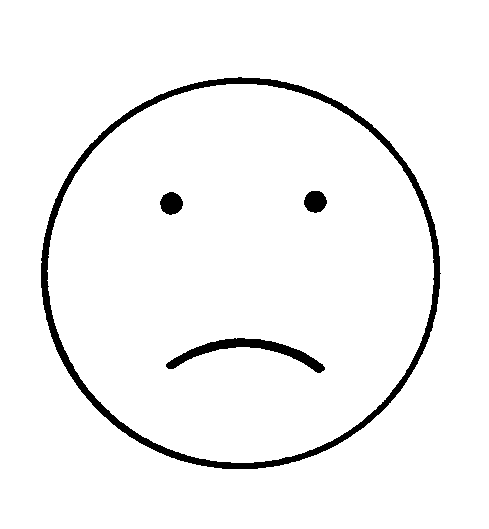** | **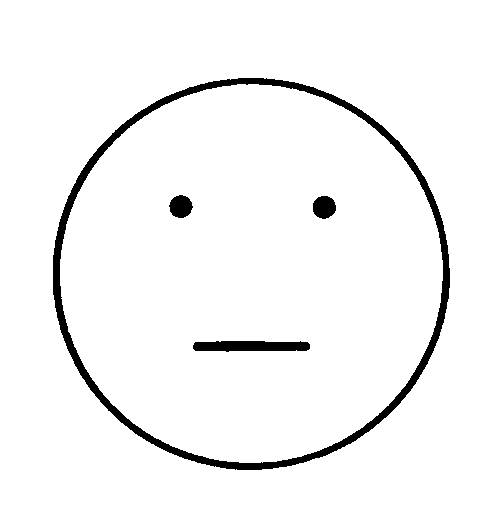** | **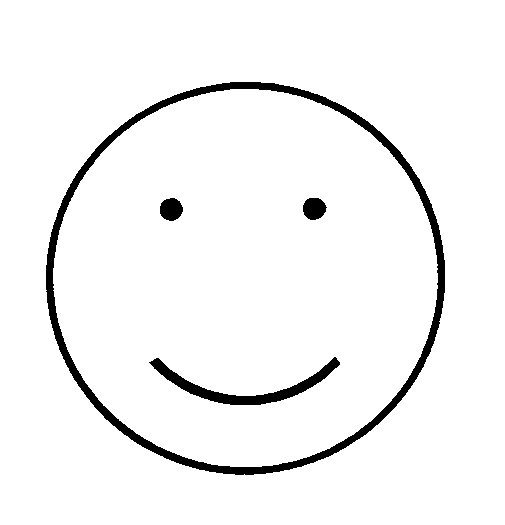** | **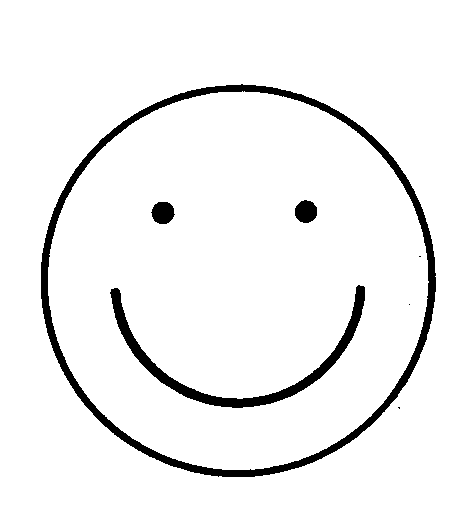** |
| --- | --- | --- | --- | --- |
| ❑  Very unhappy | ❑ | ❑ | ❑ | ❑  Very happy |

1. How happy are you with your **social connections** to the people **in your own ethnic/cultural community**?

| **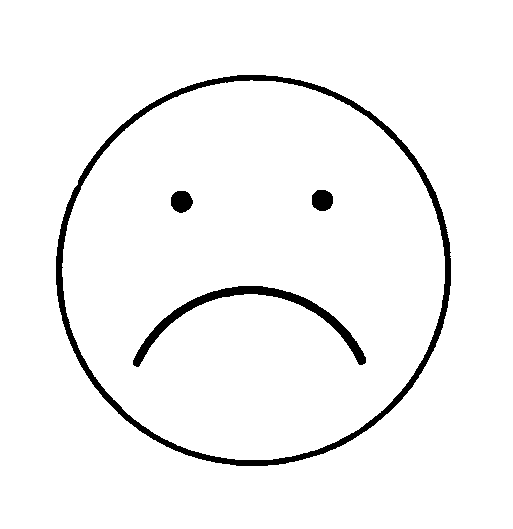** | **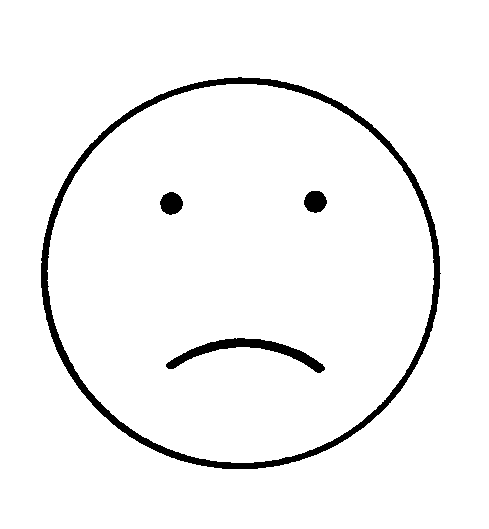** | **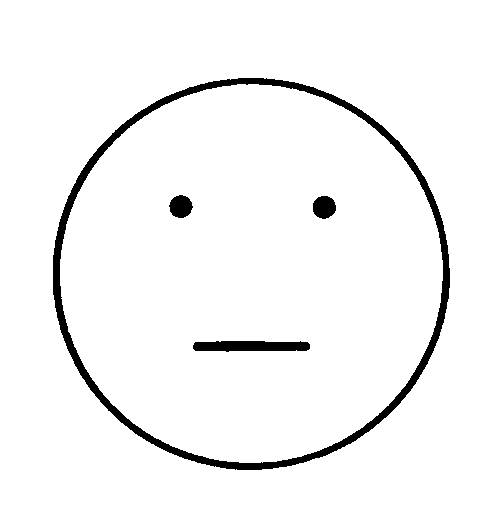** | **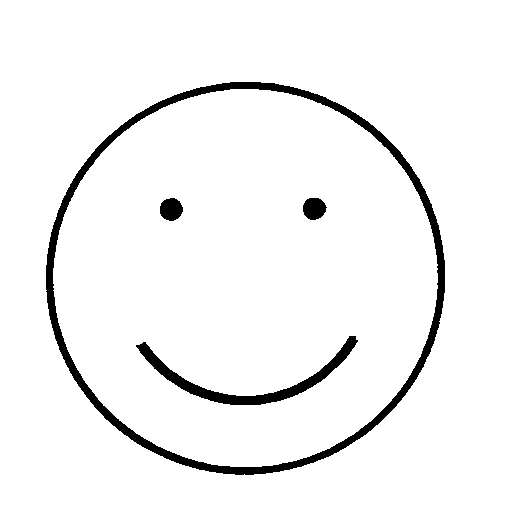** | **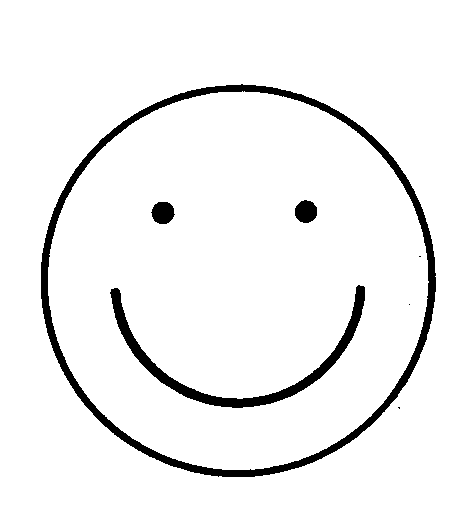** |
| --- | --- | --- | --- | --- |
| ❑  Very unhappy | ❑ | ❑ | ❑ | ❑  Very happy |

1. How happy are you with your **social connections** to **people in general**?

| **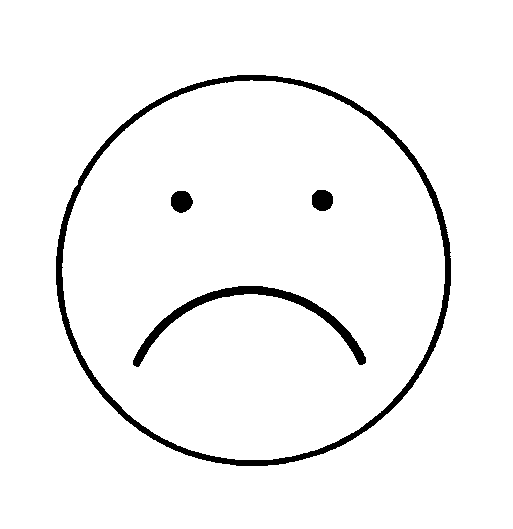** | **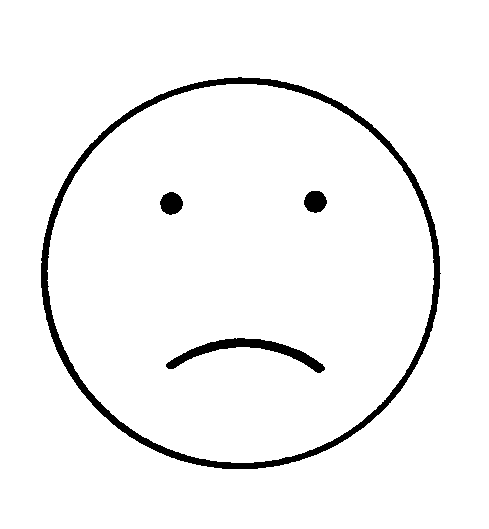** | **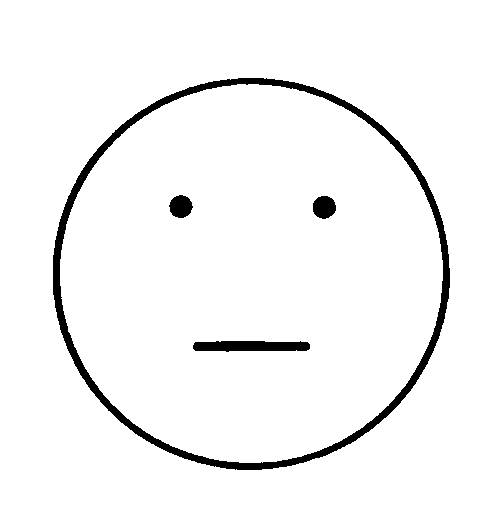** | **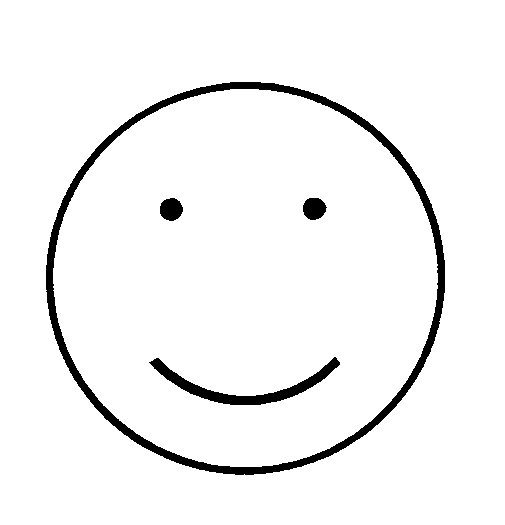** | **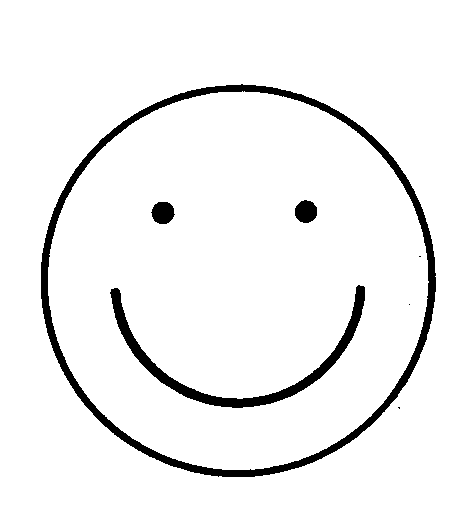** |
| --- | --- | --- | --- | --- |
| ❑  Very unhappy | ❑ | ❑ | ❑ | ❑  Very happy |

1. How **happy are you with the help and support** that you get from people that you know?

| **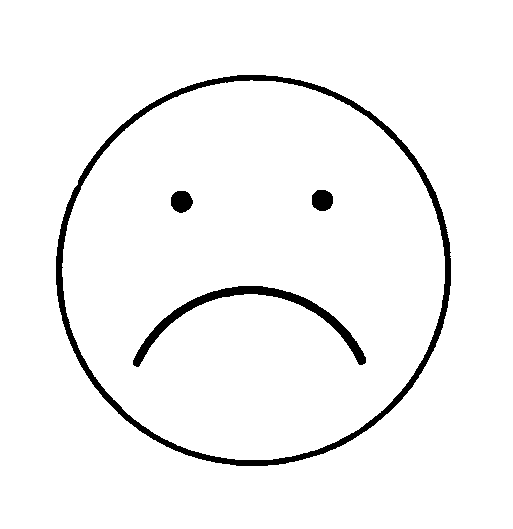** | **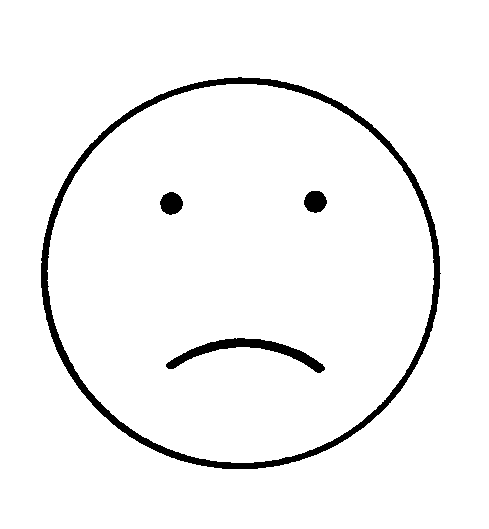** | **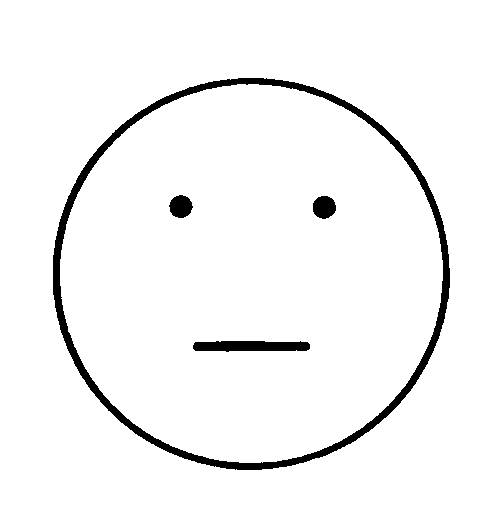** | **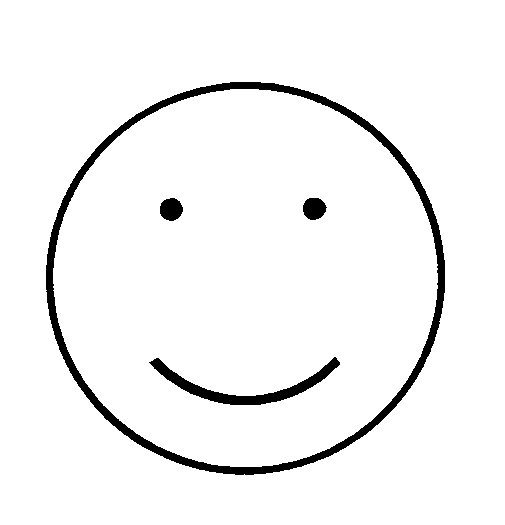** | **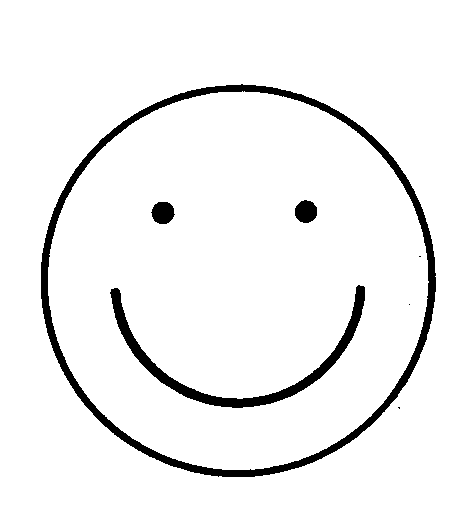** |
| --- | --- | --- | --- | --- |
| ❑  Very unhappy | ❑ | ❑ | ❑ | ❑  Very happy |

1. Overall, how often have you participated in any **unpaid volunteering** activities in Australia?

| ❑ | ❑ | ❑ | ❑ | ❑ | ❑ |
| --- | --- | --- | --- | --- | --- |
| Most days | Once a week | Several times a month | Once a month | Less than once a month | Never |

🡺 Please tell us more about these activities

1. Overall, how often have you participated in any **community groups** (such as ethnic, religious, social or sport) in Australia?

| ❑ | ❑ | ❑ | ❑ | ❑ | ❑ |
| --- | --- | --- | --- | --- | --- |
| Most days | Once a week | Several times a month | Once a month | Less than once a month | Never |

🡺 Please tell us more about these activities

1. Do you have any **caring responsibilities** in Australia?

| ❑ | ❑ |
| --- | --- |
| Yes | No |

🡺 **If yes,** please tell us more about these responsibilities

**Section 4:**

**Your Health and Wellbeing**

1. Overall, how would you rate your health over the past 4 weeks?

| ❑ | ❑ | ❑ | ❑ | ❑ | ❑ |
| --- | --- | --- | --- | --- | --- |
| Excellent | Very good | Good | Fair | Poor | Very poor |

1. Over the past 4 weeks, how much did **physical health problems** limit your usual physical activities (such as walking or climbing stairs)?

| ❑ | ❑ | ❑ | ❑ | ❑ |
| --- | --- | --- | --- | --- |
| Not at all | Very little | Somewhat | Quite a lot | Could not do physical activities |

1. Over the past 4 weeks, how much **difficulty did you have doing your daily work**, both at home and away from home, because of your **physical health?**

| ❑ | ❑ | ❑ | ❑ | ❑ |
| --- | --- | --- | --- | --- |
| Not at all | Very little | Somewhat | Quite a lot | Could not do daily work |

1. How much **bodily pain** have you had over the past 4 weeks?

| ❑ | ❑ | ❑ | ❑ | ❑ | ❑ |
| --- | --- | --- | --- | --- | --- |
| None | Very mild | Mild | Moderate | Severe | Very severe |

1. Over the past 4 weeks, **how much energy** did you have?

| ❑ | ❑ | ❑ | ❑ | ❑ |
| --- | --- | --- | --- | --- |
| None | A little | Some | Quite a lot | Very much |

1. Over the past 4 weeks, how much did your **physical health or emotional problems limit your usual social activities** with family or friends?

| ❑ | ❑ | ❑ | ❑ | ❑ |
| --- | --- | --- | --- | --- |
| Not at all | Very little | Somewhat | Quite a lot | Could not do social activities |

1. Over the past 4 weeks, how much have you been bothered by **emotional problems** (such as feeling anxious, depressed or irritable)?

| ❑ | ❑ | ❑ | ❑ | ❑ |
| --- | --- | --- | --- | --- |
| Not at all | Slightly | Moderately | Quite a lot | Extremely |

1. Over the past 4 weeks, how much did **personal or emotional problems** keep you from doing your usual work, school or other daily activities?

| ❑ | ❑ | ❑ | ❑ | ❑ |
| --- | --- | --- | --- | --- |
| Not at all | Very little | Somewhat | Quite a lot | Could not do daily activities |

1. Do you have a disability, injury or health condition that has lasted or is likely to last 12 months or more?

| ❑ | ❑ |
| --- | --- |
| Yes | No |

1. Have you **visited a doctor** or other health professional in the **last 12 months** in Australia?

| ❑ | ❑ |
| --- | --- |
| Yes | No |

1. How much do you think your **housing affects your health and wellbeing**?

| ❑ | ❑ | ❑ | ❑ | ❑ |
| --- | --- | --- | --- | --- |
| Not at all | Slightly | Moderately | Quite a bit | A great deal |

🡺Please tell us why you think this?

1. How much do you think that the **neighbourhood where you live affects your health and wellbeing**?

| ❑ | ❑ | ❑ | ❑ | ❑ |
| --- | --- | --- | --- | --- |
| Not at all | Slightly | Moderately | Quite a bit | A great deal |

🡺Please tell us why you think this?

1. Have you experienced **discrimination or been treated unfairly** in Australia because of your skin colour, ethnic origin or religion?

**please tick all that apply**

| ❑ | ❑ | ❑ |
| --- | --- | --- |
| Yes, more than 12 months ago | Yes, within the last 12 months | No 🡺go to question. 59 |

🡺 **If yes**, please tell us in which situation:

| ❑ | In my neighbourhood | ❑ | From the police |
| --- | --- | --- | --- |
| ❑ | Getting/keeping housing | ❑ | Getting a job/at work |
| ❑ | Getting help with health (such as at a hospital or doctor) | ❑ | Getting services (such as shops, bars or taxis |
| ❑ | Getting financial assistance (such as a loan or mortgage) | ❑ | School/TAFE or other educational places |
| ❑ | On public transport |  |  |
| ❑ | Other ___________________________________________________________________________________ | | |
|  |  | | |

🡺 **If yes**, please tell us about your experience

🡺 **If yes**, do you think this discrimination or unfair treatment has affected your health and wellbeing:

| ❑ | ❑ | ❑ | ❑ | ❑ |
| --- | --- | --- | --- | --- |
| Not at all | Slightly | Moderately | Quite a bit | A great deal |

1. How do you feel about your **financial situation**?

| **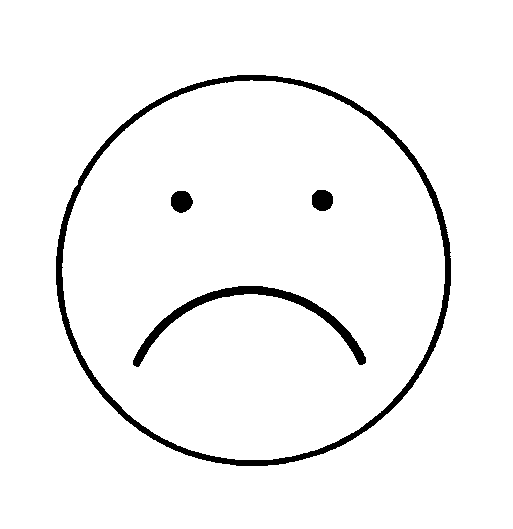** | **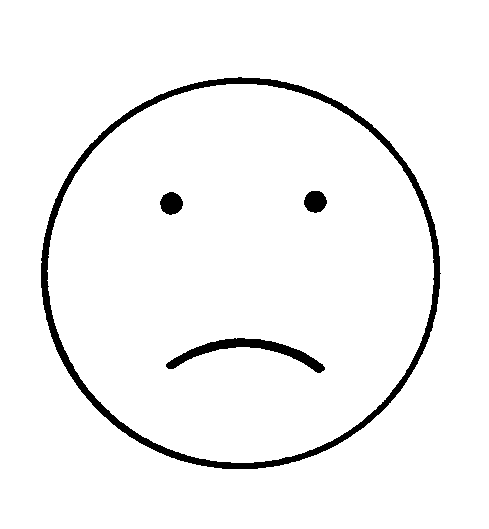** | **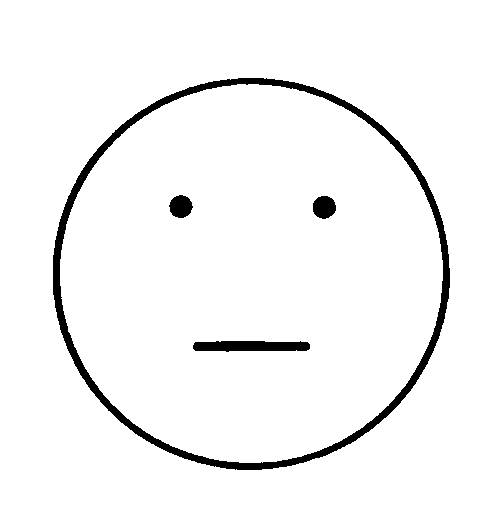** | **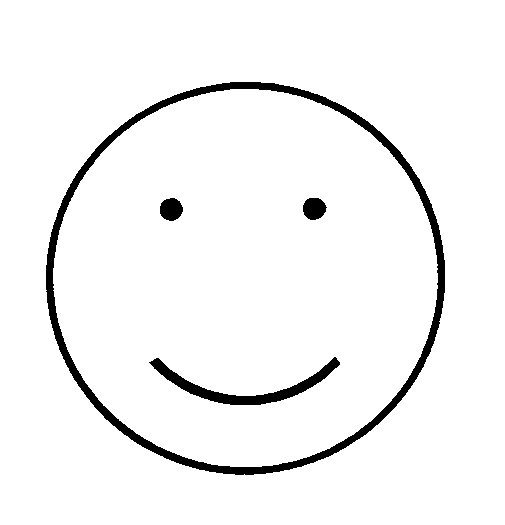** | **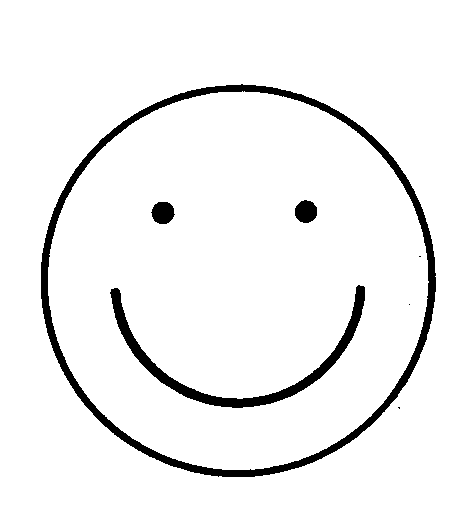** |
| --- | --- | --- | --- | --- |
| ❑  Very unhappy | ❑ | ❑ | ❑ | ❑  Very happy |

1. Has there been any time in the last 12 months in Australia that you, or members of your household, **ran out of food and couldn't afford to buy more**?

| ❑ | ❑ |
| --- | --- |
| No | Yes |

1. To what extent do you agree that most people can be **trusted**?

| ❑ | ❑ | ❑ | ❑ |
| --- | --- | --- | --- |
| Not at all | Only slightly | To a moderate extent | To a great extent |

1. To what extent do you have a **sense of belonging in Australia?**

| ❑ | ❑ | ❑ | ❑ |
| --- | --- | --- | --- |
| Not at all | Only slightly | To a moderate extent | To a great extent |

1. Please tell us how much you agree or disagree with the statement **“I feel in control of my life”**

| ❑ | ❑ | ❑ | ❑ | ❑ |
| --- | --- | --- | --- | --- |
| Disagree a lot | Disagree a bit | Don't agree or disagree | Agree a bit | Agree a lot |

1. Please tell us how much you agree or disagree with the statement **“I feel hopeful about the future”**

| ❑ | ❑ | ❑ | ❑ | ❑ |
| --- | --- | --- | --- | --- |
| Disagree a lot | Disagree a bit | Don't agree or disagree | Agree a bit | Agree a lot |

1. Overall, how **happy are you with your life in Australia**?

| **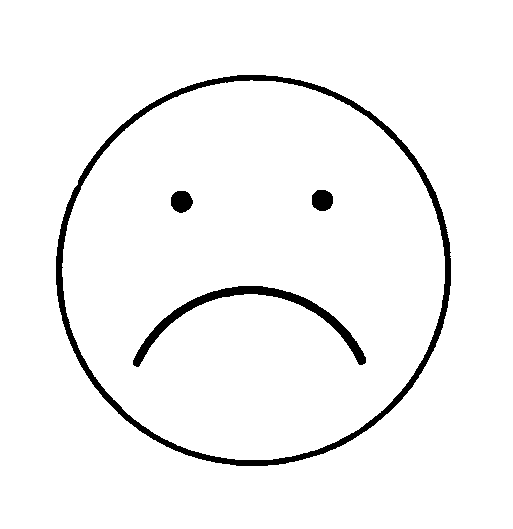** | **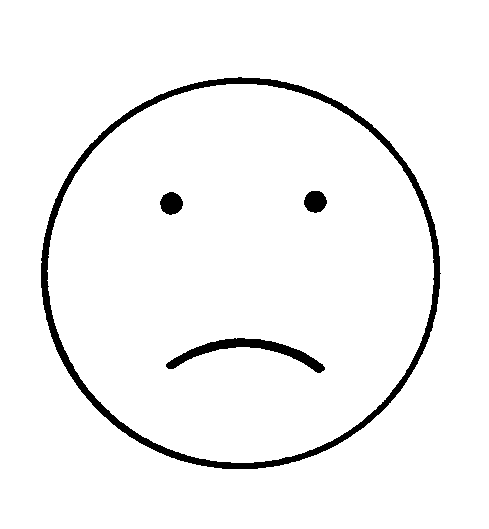** | **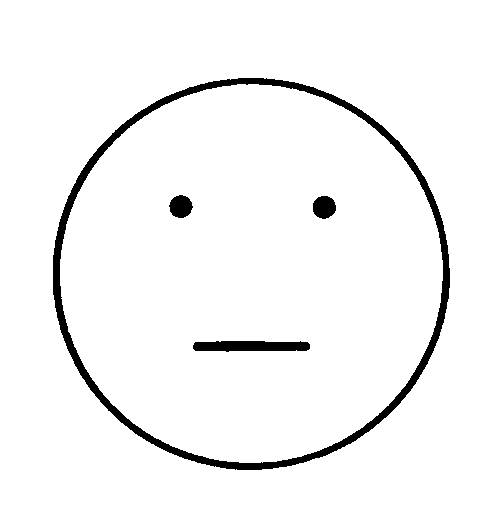** | **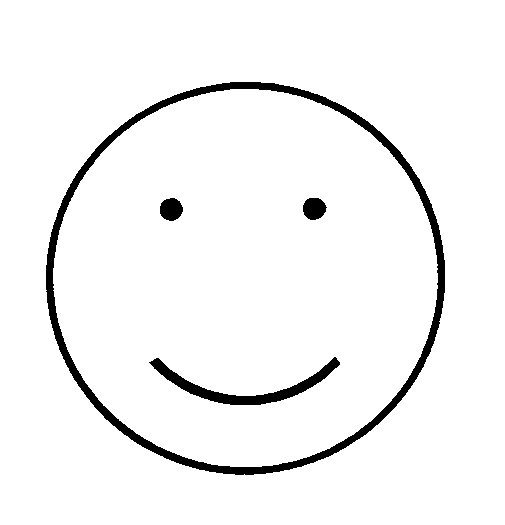** | **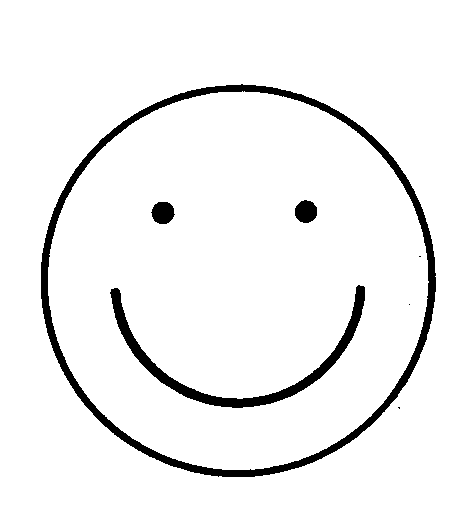** |
| --- | --- | --- | --- | --- |
| ❑  Very unhappy | ❑ | ❑ | ❑ | ❑  Very happy |

🡺Please tell us more about this in the box below.

1. Is there anything else you want to tell us about your life Australia?

Where did you get this survey from?

| ❑ | Family/friends | ❑ | Mercy Housing of Welcome |
| --- | --- | --- | --- |
| ❑ | Anglicare | ❑ | Migrant Resource Centre |
| ❑ | Australian Refugee Association | ❑ | Multicultural SA |
| ❑ | Baptist Care | ❑ | MYSA (Multicultural Youth SA) |
| ❑ | HomeStart | ❑ | Red Cross |
| ❑ | Hope’s Café | ❑ | STTARS |
| ❑ | Housing SA | ❑ | TAFE SA |
| ❑ | Life Without Barriers | ❑ | The Welcome Centre |
| ❑ | Other__________________________________________________________________________ | | |

Office Use Only:

Assisted completion: Y / N Interpreter: Y / N

Location:
