# Supplementary material for: Discrimination: a health hazard for people from refugee and asylum-seeking backgrounds resettled in Australia
Source: BMC Public Health. 2020 Jan 28;20:108. doi: 10.1186/s12889-019-8068-3 (PMC6986068; doi:10.1186/s12889-019-8068-3)
Supplement: Supplementary file 2 — Additional file 2. 'Belonging Begins at Home' semi-structured interview guide. [file 12889_2019_8068_MOESM2_ESM.docx]

**Additional file 2: ‘Belonging Begins at Home’ Semi-structured Interview Guide**

Background information (age, children, journey to Australia, time in Australia, cultural/ethnic background, religion, visa)

*Housing experiences:*

- First house in Australia – where first lived (Adelaide/interstate, suburb), satisfaction with, reasons for moving
- Current house – how found current house, how long there, how happy – why/why not,
- Future plans - any plans to move? Why?
- Past housing – how many times moved, where lived, how housing found, supports received in finding housing, what liked/didn’t like, overall satisfaction
- Current suburb/neighbourhood – experiences of, satisfaction with, previous suburbs
- What things are important in a house?
- What things are important in a neighbourhood?
- Health and wellbeing – current physical and mental wellbeing, main effects on, is anyone helping with these health issues
- Has housing affected health and wellbeing (mental and physical) - how?

*Supports in Australia:*

- Social and civic participation – socialising in neighbourhood & elsewhere, main types of interactions, who with, where, involvement in volunteering or social groups
- Support - do you feel supported and connected, by whom,
- Are you satisfied with the service providers in Australia? Why/why not?

*Experiences of discrimination:*

- Any experiences of discrimination – understandings of discrimination, examples, perceived reasons
- Response/s – initial, subsequent
- Any impacts on health and wellbeing? – in what ways?
- Know others who have experienced discrimination? - examples

*Overall*

- Overall experiences of settlement in Australia?
- How could refugees/asylum seekers be better supported – with housing, in general
